# Flagellin and GroEL mediates *in vitro* binding of an atypical enteropathogenic *Escherichia coli* to cellular fibronectin

**DOI:** 10.1186/s12866-015-0612-4

**Published:** 2015-12-18

**Authors:** Claudia T. P. Moraes, Juliana M. Polatto, Sarita S. Rossato, Mariana Izquierdo, Danielle D. Munhoz, Fernando H. Martins, Daniel C. Pimenta, Mauricio J. Farfan, Waldir P. Elias, Ângela S. Barbosa, Roxane M. F. Piazza

**Affiliations:** Laboratório de Bacteriologia, Instituto Butantan, Av. Vital Brazil, 1500 - 05503-900 São Paulo, SP Brazil; Centro de Estudios Moleculares, Departamento de Pediatría, Hospital Dr. Luis Calvo Mackenna, Universidad de Chile, Santiago, Chile; Laboratório de Bioquímica e Biofísica, Instituto Butantan, São Paulo, SP Brazil

**Keywords:** Atypical EPEC, Binding, Fibronectin, Flagellin, GroEL

## Abstract

**Background:**

Enteropathogenic *Escherichia coli* (EPEC) is distinguished mainly by the presence of EPEC adherence factor plasmid (pEAF) in typical EPEC (tEPEC) and its absence in atypical EPEC (aEPEC). The initial adherence to the intestinal mucosa is complex and mediated by adhesins other than bundle-forming pilus, which is not produced by aEPEC. Extracellular matrix (ECM) proteins of eukaryotic cells are commonly recognized by bacterial adhesins. Therefore, binding to ECM proteins may facilitate colonization, invasion and/or signaling by intestinal pathogens. Previous studies from our group demonstrated that aEPEC O26:H11 (strain BA2103) showed high binding activity to fibronectin, not shared by its counterpart, aEPEC O26:HNM.

**Results:**

In the present study, using mass spectrometry after fibronectin-associated immunoprecipitation, two proteins, flagellin (50 kDa) and GroEL (52 kDa), were identified and BA2103 binding ability to fibronectin was inhibited in the presence of anti-H11 and anti-GroEL sera, but not by either naïve rabbit or other unrelated sera. It was also observed that the presence of purified flagellin inhibits adhesion of BA2103 to cellular fibronectin in a dose-dependent manner. Additionally, BA2103 GroEL is similar to the same protein of uropathogenic *E. coli*.

**Conclusions:**

Our results suggest that flagellin may play a role in the *in vitro* interaction of BA2103 with cellular fibronectin, and GroEL can be an accessory protein in this process.

**Electronic supplementary material:**

The online version of this article (doi:10.1186/s12866-015-0612-4) contains supplementary material, which is available to authorized users.

## Background

Adherence to intestinal cells is a critical step in enteropathogenic *Escherichia coli* (EPEC) pathogenesis. EPEC expresses intimin adhesin, an adherence factor chromosomally encoded by the *eae* gene [1], which is involved in the receptors recognition, located at the surface of target cells (translocated intimin receptor – Tir, β1-integrin and nucleolin) [2, 3]. The intimin-Tir interaction plays a role in attaching and effacement lesion followed by intestinal colonization. Moreover, EPEC is distinguished by the presence of EPEC adherence factor plasmid (pEAF) in typical EPEC (tEPEC) and its absence in atypical EPEC (aEPEC) [4]. Also, lack of bundle-forming pilus (BFP) production [5, 6], presence of hemolysins [7] and autotransporter proteins [8, 9] existing in other diarrheagenic *E. coli* pathotypes characterize aEPEC [10, 11]. In tEPEC, the role of BFP either in initial contact or in bacteria-bacteria interaction is well established [12, 13]. However, in aEPEC this adhesion has been attributed to EspA and various accessory adhesins described in other pathogenic *E. coli* strains [14].

The ability to adhere to extracellular matrix (ECM) proteins has been shown to be essential for the virulence of several pathogens [15]. The ECM proteins comprise a diverse group that function as a barrier, support for epithelial cells, and are responsible for development, growth, and maintenance of mammalian tissues [16]. The composition of ECM differs in various organs, but fibronectin, collagen types I to XV, and laminin are common constituents [17]. ECM proteins are commonly recognized by bacterial adhesins and have been shown to act as substrates for bacterial adherence to eukaryotic cells [15, 18–22]. Enteric bacterial pathogens can interact with ECM either during inflammation or in the tight junctions opening [23]. Therefore, binding to ECM proteins may facilitate colonization, invasion, and/or signaling by intestinal pathogens [17]. Fibronectin is an ECM molecule targeted by several pathogens and is formed by dimers covalently linked by a pair of disulfide bonds near their carboxyl termini [24]. Fibronectin is responsible for connecting the collagen scaffold and other ECM components [22], and was the first eukaryotic cell receptor described for bacteria [18].

Recently, some conserved proteins, such as outer membrane protein A (OmpA), flagellin (FliC) and *E. coli* GroEL have been described as involved in adhesion, colonization, invasion and dissemination or as major antigens in many important pathogens [25–31]. FliC, the subunit of flagellum structure, contains highly conserved N- and C-termini, while its central region is significantly variable and provides antigenic differences [32]. FliC is involved in motility and pathogenesis [33–36], and also can interact with cell surface polypeptide receptors on monocytes and activate Toll-like receptors 5 (TLR-5) [37]. GroEL is a multitask protein, which function as a prototypical and indispensable molecular chaperone in stress survival. In addition, this protein presents moonlighting activities acting as a cell surface receptor for various pathogens ligands. Between 250–300 *E. coli* proteins bind to GroEL, 85 of them are obligate client to GroEL [38].

Previous results from our group demonstrated that a subset of atypical EPEC presented ability to bind to immobilized ECM proteins. Among them, the O26:H11 (BA2103) strain consistently presented the highest binding ability to cellular fibronectin [7]. Taking these results in consideration, we investigated in the current study putative candidates of this particular strain that might mediate binding to cellular fibronectin. Our results indicate that flagellin may play a role in the *in vitro* interaction with this ECM component, and GroEL can be an accessory protein in this process.

## Methods

### Bacterial strains

Atypical EPEC (aEPEC) strains BA2103 (serotype O26:H11) and 2271-1/85 (serotype O26:HNM, where NM indicates nonmotile) were previously characterized by the *eae*+/EAF-/*bfpA*- genetic profile, lack of production of BFP and production of the localized adherence-like (LAL) pattern on HEp-2 cells [5, 6, 39]. Molecular typing of *fliC* defined the H type of 2271-1/85 strain as H11 [39]. Binding ability to ECM of supernatant proteins from BA2103 was described elsewhere [7]. A non-pathogenic K12 *E. coli* DH5α was included as a control strain [40]. Also, twenty O26:H11 aEPEC strains were employed in the fibronectin binding screening [5, 39]. This study does not involve humans, human data or animals, since all strains employed herein belong to the Bacteriology Laboratory bacterial collection of Butantan Institute, São Paulo, SP, Brazil.

### Binding of *E. coli *strains to fibronectin

In order to confirm the binding ability of BA2103 strain to fibronectin and compare it to 2271-1/85 and DH5α strains, 40 μL of bacteria culture growth (1.5 × 10^8^) were incubated in ELISA microplates previously coated with 1 μg of cellular fibronectin, or 1 μg of bovine serum albumin (BSA) (Sigma Chemical Co.) at 37 °C for 4 h [41]. Also, 40 μL of serially diluted bacterial suspensions (from 10^8^ to 10^5^) were tested. An additional assay was done preincubating 10^7^ bacterial cells with increasing concentrations of cellular fibronectin (from 0.2 to 25.6 μg) followed by incubation in ELISA microplates previously coated with 1 μg of cellular fibronectin. Quantification of adhered bacteria was determined by removing the bacteria adhered to fibronectin and BSA with PBS containing 0.05 % Triton X-100. Serial dilutions were plated onto Luria Bertani (LB) agar plates and the number of bacteria was determined by counting colony-forming units (CFU) [20]. CFU values were obtained from triplicates of four independent experiments.

Additionally, the binding ability to cellular fibronectin of 20 strains belonging to the O26:H11 serotype was accessed employing the crystal violet staining measurement as described elsewhere [42]. Experiments were performed in duplicate of two independent experiments and the average values were calculated.

### Protein identification

Supernatant proteins were obtained by growing BA2103 in 50 mL of TSB at 37 °C for 18 h (150 rpm), followed by centrifugation at 10,000 *g* for 15 min. This supernatant was filtered through a 0.22 μm membrane and protein concentration was determined by a Bradford assay. Protein identification was achieved by matrix-assisted laser desorption ionization–time of flight mass spectrometry (MALDI-TOF) analyses. Prior to MALDI-TOF analyses, 100 μg of supernatant proteins from BA2103 were incubated with 25 μg/mL of fibronectin at 25 °C for 90 min. Fibronectin-associated proteins were obtained by immunoprecipitation analysis using A/G agarose columns (Pierce) conjugated with anti-fibronectin antibodies (Sigma). Proteins recovered from the column were visualized by silver-stained SDS-PAGE, and protein bands were excised from SDS gel for MALDI-TOF analyses (Mass Spectrometry Core Laboratory, University of Texas Medical Branch).

### Sequence alignments of flagellin and GroEL from *Escherichia coli* O26:H11 strains

Amino acid **s**equences of flagellin (gi|260855903) and GroEL (gi|18028158) obtained by MALDI-TOF analyses were compared to other related sequences available in GenBank database. All sequence alignments and analyses were performed with Basic Local Alignment Search Tool (BLAST) program (http://blast.ncbi.nlm.nih.gov/BlastAlign.cgi), MUSCLE [43] and BioEdit Sequence Alignment Editor vs 7.2.5.0.

The *fliC* gene of BA2103 was amplified by PCR and sequenced using primers described elsewhere [44] in order to investigate minor nucleotide alterations. Three additional internal primers (sequence 1 *fliC* (R): GCCTGACCTGCTGCG; sequence 2 *fliC* (R): CACTGACTTACCATC; sequence 3 *fliC* (F): CATGTCTCGTGCG) were designed to obtain full-length *fliC* sequence.

### H11 flagellin purification

Flagellin produced by BA2103 (O26:H11) was extracted from 50 mL cultures as described elsewhere with some modifications [45]. Briefly, bacterial cells were collected by centrifugation, suspended in 2 mL of phosphate-buffered saline (pH 7.4) and sheared for 2 min in bench mixer (Genie 2) at maximal speed. Cells were kept on ice bath for 1 min and the procedure was repeated three times. The cell suspension was then centrifuged at 10,000 X g for 10 min to remove the bacterial cells. Culture supernatants containing the sheared flagellin were precipitated with acetone. The resulting pellet was suspended in PBS and, finally, submitted to heat treatment (65 °C for 30 min) to depolymerize filaments into flagellin monomers. Protein concentration was determined using BCA assay (Pierce) and purity was checked by SDS-PAGE and immunoblotting using anti-H11 serum. Also an ELISA was employed in order to check anti-H11 reactivity to purified flagellin.

### Detection of flagellin and GroEL in *E. coli* strains by immunofluorescence

The presence of flagellin and GroEL was evaluated in three bacterial strains (BA2103, 2271-1/85 and *E. coli* DH5α) by immunofluorescence employing anti-H11 and anti-GroEL sera. *E. coli* strains were cultivated at 37 °C in LB broth for 16–18 h, and a smear from the bacterial pellets was fixed with 4 % paraformaldehyde. Slide glasses with fixed bacteria were washed with phosphate-buffered saline (PBS 0.01 M, pH 7.4) and blocked with 10 % goat serum in PBS (PBS-GS) at 25 °C for 1 h, followed by incubation at 25 °C for 2 h with rabbit anti-GroEL (1:500) or rabbit anti-H11 (1:200) antibodies diluted in PBS-GS and goat anti-rabbit IgG conjugated with FITC diluted 1:250 or 1:100, respectively at 25 °C for 1 h. Between incubations the reactions were washed twice with PBS, and then slide glasses were mounted with glass coverslips. Bacteria were visualized on Axioskop fluorescence microscope (Zeiss, Germany), with a 400X magnification.

### Binding inhibition to fibronectin

In order to validate the interaction of flagella and GroEL with cellular fibronectin, an *in vitro* inhibition assay was performed. BA2103 was cultivated in LB broth and 1.5 x 10^7^ of bacteria suspension was preincubated with serial dilutions of rabbit anti-H11 or rabbit anti-GroEL (1:500 to 1:8,000) (Sigma-Aldrich) antibodies at 37 °C for 2 h, at 50 rpm in a platform shaker. Bacteria were then incubated at 37 °C for 4 h in ELISA microplate wells previously coated with 1 μg of cellular fibronectin. Serial dilutions (1:500 to 1:8,000) of naïve rabbit serum, rabbit anti-H9, anti-human vitronectin (Sigma-Aldrich), anti-human C1q and anti-human C3 were employed as negative controls of binding inhibition assay. An additional assay was done by incubating increasing concentrations of purified flagellin (7.8 to 500 μg) in ELISA microplate wells previously coated with 1 μg of cellular fibronectin, followed by an incubation of bacteria culture growth (1.5 x 10^7^). Quantification of adhered bacteria was achieved by removing them from microplate wells with PBS containing 0.05 % Triton X-100. Serial dilutions were plated onto LB agar plates and CFU was determined as described elsewhere [20]. CFU values were obtained from triplicates of four independent experiments in the presence or absence of antisera or in the presence or absence of purified flagellin.

### Statistical analyses

The colony-forming units (CFU) and absorbance values were analyzed by Graph Prism® 5.01, using unpaired Student’s *t*-test. The differences were considered statistically significant when *p* ≤ 0.05.

## Results

### Binding of *E. coli* strains to cellular fibronectin

**Fig. 1:**
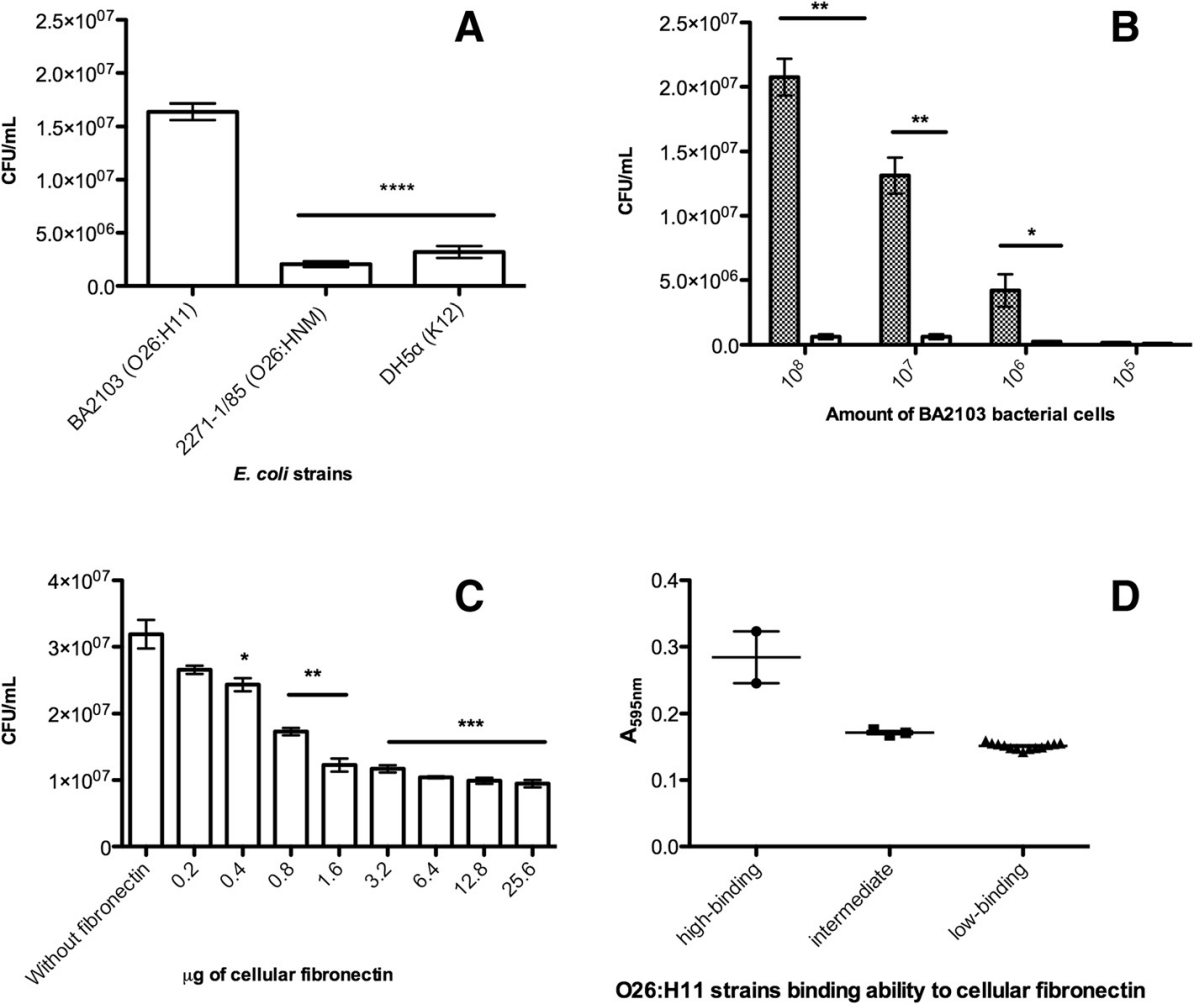
Binding assay: **a** Binding to fibronectin of 40 μL of bacteria culture growth of BA2103, 2271-1/85 and DH5α strains incubated in ELISA microplates previously coated with cellular fibronectin, or BSA. **b** Serial dilutions of BA2103 incubated in ELISA microplates previously coated with cellular fibronectin (hatched bars) or BSA (white bars). **c** BA2103 preincubated with increasing concentrations of cellular fibronectin prior to incubation in ELISA microplates previously coated with cellular fibronectin. Adhered bacteria were recovered with 0.05 % triton-X100 in PBS and plated on LB agar in serial dilutions and then the colony-forming units were counted. Differences were statistically significant comparing the binding to fibronectin of BA2103 and the other two strains (*p* < 0.0001) and comparing the binding of BA2103 to fibronectin or to BSA independent of bacteria concentration (*p* < 0.004). It was also statistically significant compared the preincubation in absence of fibronectin or in presence of dose-dependent of fibronectin (from *p* < 0.03 to 0.0005). **d** O26:H11 strains screened by binding ability to cellular fibronectin employing the crystal violet staining measurement. Absorbance’s means between high and low binding groups were statistically significant (*p* < 0.0001, R^2^ 0.9025) also means difference between high and intermediate were statistically significant (*p* = 0.0310)

The binding ability of BA2103 to cellular fibronectin was compared to another aEPEC strain, a nonmotile O26 (2271-1/85) and a nonmotile K12 *E. coli* strain. The 2271-1/85 strain differs from BA2103 in terms of lack of motility and flagellin production. The binding of BA2103 to fibronectin was much more pronounced and this difference was statistically significant compared to the binding of the other two strains to this particular ECM component (Fig. 1a). BA2103 bound to cellular fibronectin in a dose-dependent manner and this interaction was specific, since no adhesion to BSA was observed in any dilutions of bacterial suspensions (Fig. 1b). Also a competitive dose-dependent and saturable inhibition was observed in preincubation of BA2103 bacterial cells with cellular fibronectin prior to binding to immobilized fibronectin (Fig. 1c).

Furthermore, twenty aEPEC O26:H11 strains were screened by their binding ability to cellular fibronectin by crystal violet staining. Employing this method we were able to arbitrary separate three groups according to their ability to bind to cellular fibronectin: high-binding strains, including BA2103 and BA2459, and low-binding strains, including non-motile 2271-1/85. The statistical analyses showed that the absorbance’s means between both groups were statistically significant (*p* < 0.0001, R^2^ 0.9025) and also the variances between both groups were significant (*p* < 0.0001). The third group presenting intermediate binding ability, comprised by three strains (0791-1/85; 4851-3/86 and IC50) (Fig. 1d).

### Identification of proteins involved in the recognition of cellular fibronectin by BA2103

**Fig. 2:**
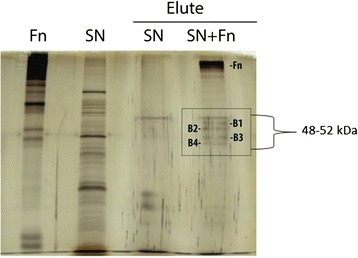
Co-immunoprecipitation of fibronectin-bound proteins. 100 μg of supernatant proteins (SP) were incubated with 25 μg/mL of Fn (Sp + Fn) or PBS (SP + PBS) and the mixture was added to a column with anti-Fn antibodies for co-immunoprecipitation analysis. Immunoprecipitated proteins were visualized by silver stained SDS-PAGE and protein bands (B1, B2, B3 and B4) were excised for MALDI-TOF analyses

Employing fibronectin-associated immunoprecipitation, four proteins were identified by mass spectrometry (Fig. 2, Table 1). Two of them, flagellin (giI260855903 – 50 kDa) and GroEL (giI18028158 – 52 kDa) with 2.51E-43 and 1.26E-22 of expectation score, respectively, could be considered as putative adhesins for bacterial cell interaction.

**Table 1:** Identification of fibronectin-associated proteins by MALDI-TOF analyses (**p value = 0,05 and Protein Significance Score = 71)

Protein	Accession	Average Mass (kDa)	^a^AAs	E-values
flagellin (*Escherichia coli* O26:H11 str.11368)	gi|260855903	50.9	484	2.51E-43
GroEL (*Escherichia coli*)	gi|18028158	52.0	277	1.26E-22
lysine-tRNA ligase (*Escherichia coli*)	gi|486212064	57.7	337	1.26E-28
protein S1	gi|223404	61.1	158	9.98E-11

^a^Availability of aminoacids

**Protein score is -10*Log(P), where P is the probability that the observed match is a random event. Protein scores greater than 62 are significant (*p*<0.05). Protein scores are derived from ions scores as a non-probabilistic basis for ranking protein hits

*In silico* analysis were performed in order to investigate which features of BA2103 flagellin or GroEL would confer their binding activities to cellular fibronectin. Multiple alignments of flagellin sequences from O26:H11 strains showed that these proteins are highly conserved, and no sequence unique to aEPEC BA2103 flagellin (FliC_BA2103_) was observed (Additional file 1: Figure S1). Considering that hypervariable domains of flagellin are involved in bacterial adhesion [46], we hypothesize that fibronectin-binding sites could be located on this region of the FliC_BA2103_. To test this, we performed an alignment by BLASTp of this region (residues 177 to 394) and fibronectin-binding domain of the *Staphylococcus aureus* FnBPA, a well-characterized fibronectin-binding protein [47]. We found a 14-amino acid sequence (YDVGGDAYTVNVDS) showing 64 % similarity to two motifs of FnBPA, which could be a putative Fn-binding site within FliC_BA2103_ (Additional file 2: Figure S2).

As demonstrated by MALDI-TOF, GroEL of BA2103 is similar to GroEL of *E. coli* J96 sequence. The results from GroEL alignment sequence showed 65 amino acids variation at equatorial domain 1 between J96 and 11368 O26:H11 strain (Additional file 3: Figure S3). We presented the alignment comparison with only one of *E. coli* O26:H11 (strain 11368), since previous analysis comparing more than 50 sequences, including different *E. coli* serotypes of H11, H7 and H6, showed 100 % of identity (data not shown). Although the sizes of compared proteins were different, hydrophobic domain at apical domain (amino acids 191–203) is conserved among all analyzed sequences (Additional file 3: Figure S3). Another important feature is that GroEL of J96 is more similar to the sequence found in *Shigella sonnei* (90,8 %) than most of *E. coli* GroEL (88.3 %) (Table 2).

**Table 2:** GroEL Sequence Identity Matrix values generated after alignment by ClustalW among *E. coli* J96 O4:K6, *Shigella sonnei* 3233-85 and *E. coli* 11368 O26:H11

Isolate	J96	3233-85	11368
J96	ID	0.908	0.883
3233-85	0.908	ID	0.972
11368	0.883	0.972	ID

**Fig. 3:**
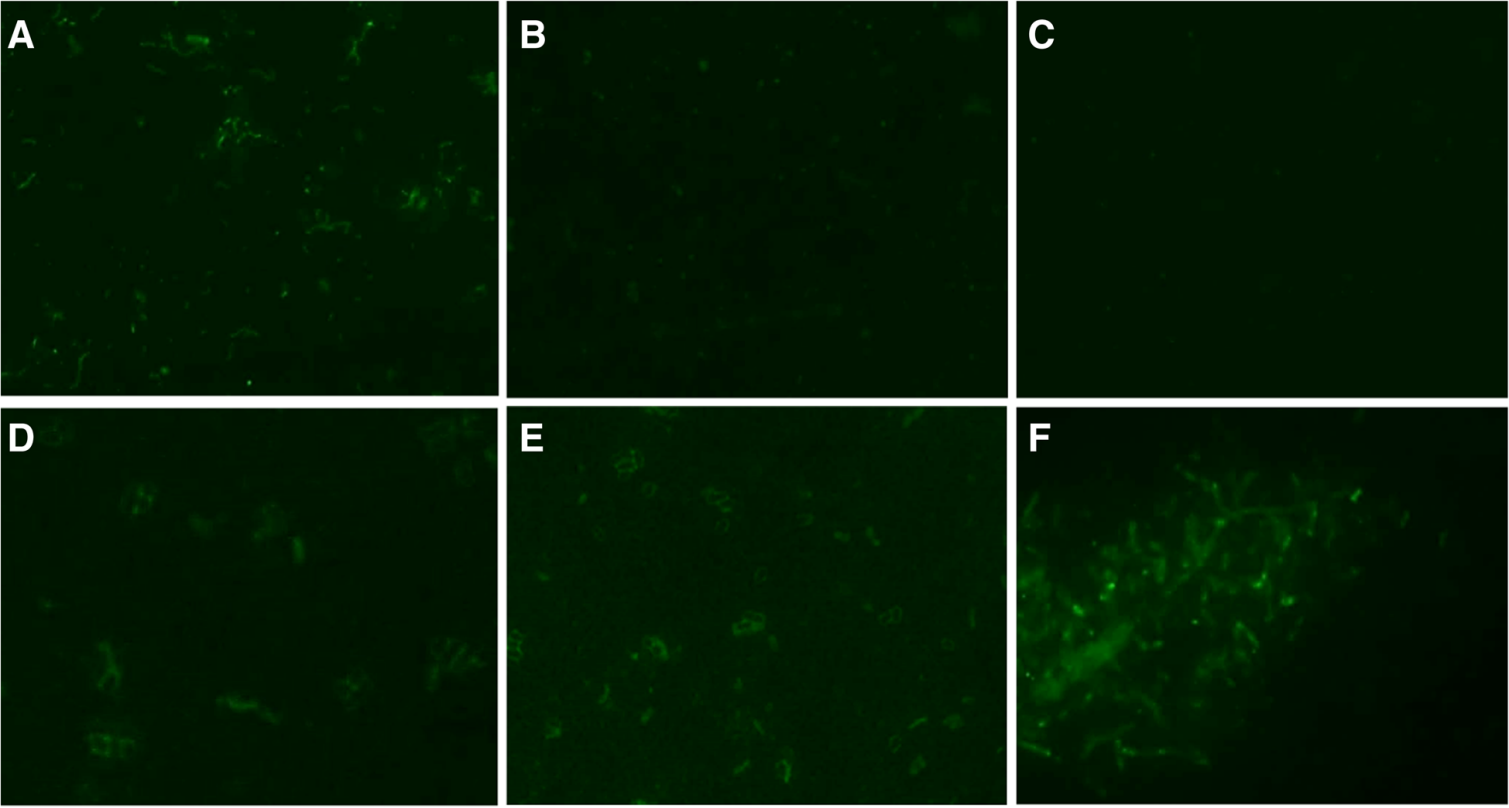
Immunofluorescence assay. Bacterial pellets from aEPEC BA2103 (panels **a** and **d**), aEPEC 2271-1/85 (panels **b** and **e**) and K12 DH5α (panels **c** and **f**) were fixed with 4 % paraformaldehyde on slide glasses and incubated with rabbit anti-H11 (panels a to c) or with rabbit anti-GroEL (panels **d** to **f**) followed by an incubation with goat anti-rabbit IgG conjugated to FITC. The immunoassay with intact, non-permeabilized bacteria was visualized on Axioskop fluorescence microscope with a 400X magnification

We also performed an immunofluorescence assay in order to assess surface localization of the two above-mentioned proteins. Employing the rabbit anti-H11 serum, flagellin was detected only in BA2103. As expected anti-H11 did not react with non-motile aEPEC (2271-1/85) and non-motile K12 DH5α (Fig. 3a, b and c). On the other hand, as GroEL is a highly conserved protein, it was detected in intact and non-permeabilized *E. coli* strains, i.e., BA2103, 2271-1/85 and DH5α (Fig. 3d, e and f).

### The role of flagellin and GroEL in the *in vitro* interaction of BA2103 to cellular fibronectin

**Fig. 4:**
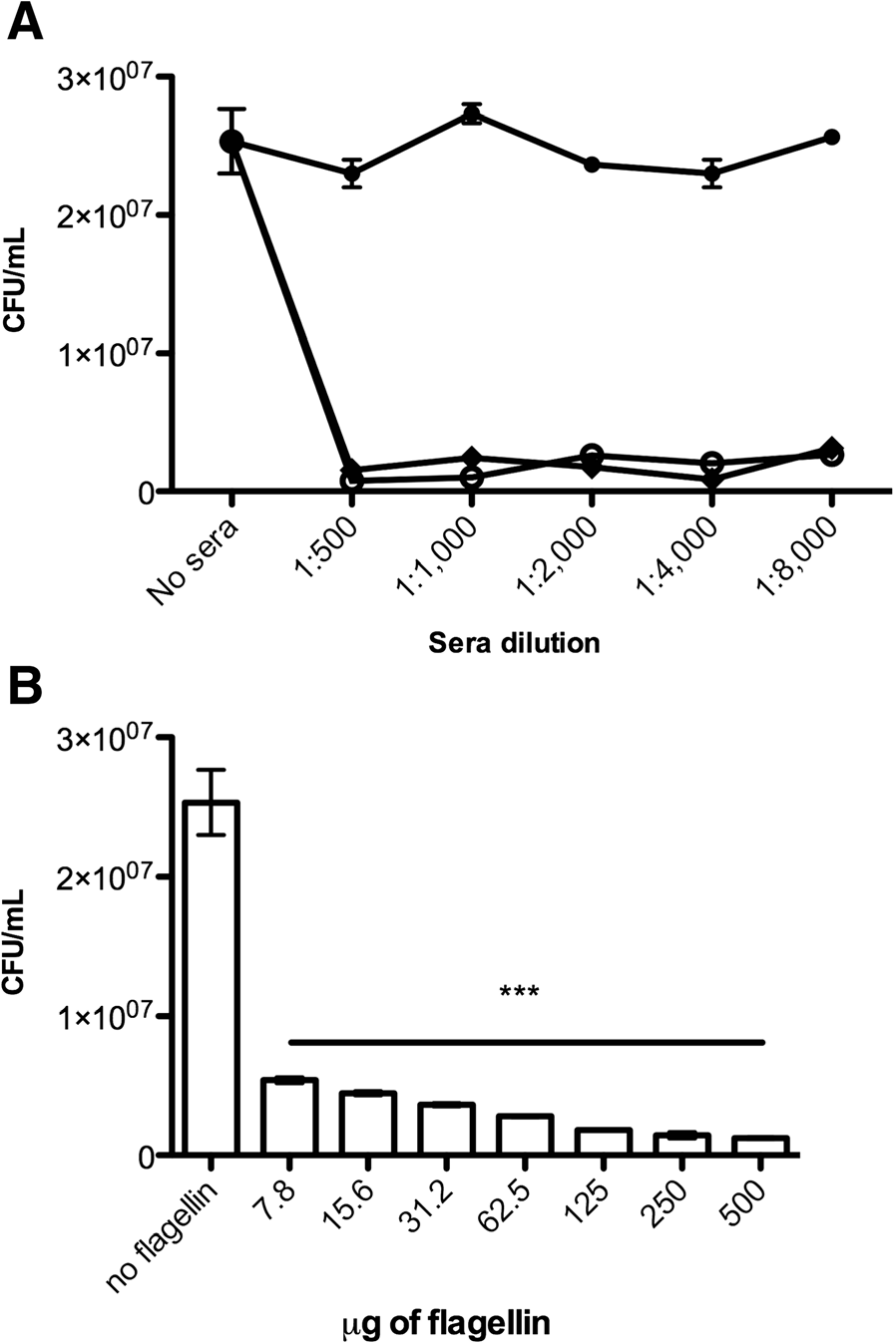
Inhibition of binding to fibronectin in the presence or absence of sera or competition with purified flagellin. **a** Naïve (•), anti-H11 (◆) or anti-GroEL () rabbit sera were serially diluted and preincubated with BA2103 and then incubated in the presence of fibronectin. **b** Different concentrations of purified flagellin were incubated in ELISA microplates previously coated with cellular fibronectin, followed by incubation with BA2103. Adhered bacteria were recovered with 0.05 % triton-X100 in PBS and then plated on LB agar in serial dilutions and then the colony-forming units were counted. Differences were statistically significant compared to the binding of BA2103 to fibronectin, in presence of naïve rabbit or anti-H11 serum (*p* = 0.0008) or anti-GroEL (*p* = 0.0008). Also significant differences were observed in presence purified flagellin (from *p* = 0.0010 to 0.0005)

To further demonstrate the role of both proteins in the interaction of BA2103 to cellular fibronectin, we performed a fibronectin binding inhibition assay using specific antibodies against H11 and GroEL proteins. Both antisera were able to strongly inhibit the binding of BA2103 to fibronectin *in vitro* independent of sera dilution. These inhibitions either with rabbit anti-H11 or anti-GroEL sera (*p* = 0.0008) were statistically significant when compared to the binding of BA2103 to fibronectin in sera absence and also when we employed naïve rabbit serum which showed no binding inhibition (Fig. 4a). Also, no binding inhibition was observed when we employed rabbit anti-H9 serum or a set of unrelated sera, such as anti-human vitronectin, anti-human C1q and anti-human C3 (data not shown).

A competitive inhibition assay between BA2103 and flagellin was done by incubating a fixed concentration of cellular fibronectin with increasing concentrations of purified flagellin (Additional file 4: Figure S4) followed by incubation with BA2103 bacterial cells. These results corroborate that flagellin may mediate *in vitro* binding of BA2103 to cellular fibronectin; since flagellin was able to block in a dose-dependent manner the subsequent interaction of BA2103 bacterial cells to cellular fibronectin (Fig. 4b).

## Discussion

Bacteria can produce proteins that interact with ECM components. These proteins are qualified as MSCRAMMs (microbial surface components recognizing adhesive matrix molecules) [22]. Thus, ECM proteins can play a bridging role between bacteria and host cells, contributing to activation of signal transduction pathways and control of later steps of pathogenesis [20]. In addition, bacterial colonization and invasion is related to laminin and fibronectin adherence in epithelial cells [17].

The high adhesiveness ability of the O26:H11 strain to ECM components, mainly fibronectin and laminin [7], prompted us to characterize *in vitro* binding ability of BA2103 to fibronectin with the aim of characterizing putative bacterial proteins involved in this interaction. Our results confirmed the binding of BA2103 bacterial cells to fibronectin. This kind of interaction has been described for enteroaggregative and enterohemorrhagic *E. coli* fimbrial adhesins [20, 21]. In order to identify which proteins could be involved in this process, fibronectin-associated immunoprecipitation was performed.

Our initial thought was that hemolysins could be partially involved in these interactions, since high levels of binding were observed with bacterial supernatant from the strains harboring *ehly*, *ehx* and *sheA* genes [7]. Therefore, we employed bacterial supernatants for fibronectin-immunoprecipitation in the present study. Surprisingly, no hemolysins were identified; on the other hand, after MALDI-TOF analyses four proteins were identified, three of them (lysine tRNA ligase, GroEL and H11 flagellin) present in *E. coli*. Despite the fact that, one cannot exclude the functions of tRNA ligase as a moonlighting protein as previously showed for glycil-tRNA [48]. It is worth to mention that flagellin is abundant in *E. coli* and is certainly present both in the bacterial membrane and in the supernatant due to physical rupture during cultivation. The same can be applicable for GroEL, considered a moonlighting protein and present not only in the cytoplasm but also on the bacterial membrane and extracellularly [38]. Thus, we decided to focus on the role of flagellin and GroEL.

It has been described that flagella not only contribute to bacterial motility but are also involved in biofilm formation, binding to host proteins, adherence, invasion, and colonization of host cells [25, 33, 36, 49–51]. Concerning EPEC, the detected binding of H6 flagella, purified from the prototype tEPEC E2348/69, to ECM components was in a dose-dependent manner to collagen and with less affinity to laminin and fibronectin, but not to vitronectin. Furthermore, the prototype tEPEC E2348/69, but not *fliC* mutant bound to ECM proteins [35]. Also, FliC in aEPEC serotype O51:H40 plays a role in adhesion, invasion and IL-8 production [36].

In our study, both aEPEC strains belong to the serotype O26:H11 but only the motile BA2103 produce flagellin and interact with fibronectin, leading us to attribute a role for the H11 flagellin in mediating bacterial binding to fibronectin. Thus, the interaction between flagellin and fibronectin may contribute to aEPEC BA2103 efficiency in tissue colonization, indirectly demonstrated herein by a significant inhibition of the binding of aEPEC binding to fibronectin in presence of different dilutions of rabbit anti-H11 serum, but not to naïve rabbit serum. Furthermore, purified flagellin (H11) was able to block in a dose-dependent manner the subsequent interaction of BA2103 bacterial cells to cellular fibronectin.

In an attempt to characterize BA2103 flagellin features that confer its binding to cellular fibronectin, *in silico* analysis was performed. No fibronectin-binding domain has been described in flagellin so far. Despite that, our data demonstrate a specific interaction between FliC_BA2103_ and fibronectin, suggesting the presence of binding sites for this ECM molecule. As hypervariable domains of flagellin bear adhesive properties [46], we selected this specific region of FliC_BA2103_ to search for Fn-binding sites, identifying a 14-amino acid residue similar to two motifs of *S. aureus* FnBPA involved in interactions with fibronectin [47]. The YDVGGDAYTVNVDS identified sequence could be a putative Fn-binding site, although specific experiments such as peptide array and site-directed mutagenesis need to be performed for mapping the Fn-binding sites within FliC_BA2103_.

GroEL was described as a conserved and immunodominant protein in *Brucella* and avian pathogenic *E. coli* [52, 53]. Besides its classical function as a chaperone, a role as adhesin- or invasin-mediating factor has been described for different pathogens such as *Mollicutes*, *Salmonella* Typhimurium, *Mycobacterium bovis* and *E. coli* [54–56]. Moreover, it has been shown that GroEL homolog of *E. coli*, the 60-kDa-heat shock protein, is not only intracellularly located but also associated with cell surface [57]. Our immunofluorescence data indicate that GroEL was also found to be associated with cell surface in all three tested strains. In aEPEC BA2103 anti-GroEL serum significantly reduced its binding capacity to fibronectin, which further supports a function for this protein as one adhesin, since no reduction was observed when unrelated sera were employed. The folding activity of GroEL on substrate proteins is dependent of its ATP induced conformational change [58]. There are three important domains described for this protein: apical, intermediate and equatorial 1 and 2 [30]. The hydrophobic binding is found on apical domain [59] and the flexibility of this site of protein facilities the binding with substrates, GroES (co-chaperonin) and non-natives polypeptides [30]. In our results, the MALD-TOF analysis identified a GroEL of BA2103 O26:H11 similar to GroEL to uropathogenic *E. coli* J96 [60]. When compared to the majority of the H11 FliC *E. coli* subtype sequences in GenBank, a difference of 65 amino acids was verify between them at Equatorial domain 1. Although no changing has been observed at the conserved binding site, a significant size alteration can affect conformation of GroEL. As the function of GroEL is folding related, conformational changes could improve exposition of binding site and higher the binding to fibronectin, consequently. This interaction would facilitate the interaction between this ECM and others *E. coli* proteins [38]. Nevertheless, structural analyses are necessary to elucidate this mechanism.

The role of flagellin and GroEL in the *in vitro* interaction of aEPEC BA2103 to cellular fibronectin herein demonstrate cannot be extended to all O26:H11 aEPEC strains. In fact, among the collection of twenty O26:H11 strains tested in this study, only BA2459 presented the same binding ability, as previously described [7]. The non-motile strains were classified in the low-binding group and it is worth to mention that among these strains, three harbor the *ehxA* gene (O26 TR EPM; 3451-3/86 and 2012–1) [39].

A frequent attribute among aEPEC strains is the finding of specific phenotypes or putative virulence factors produced by subgroups, sometimes one strain belonging to the same serotype [9, 36, 61, 62]. Adhesion, invasion, and IL-8 production in cultured intestinal epithelial cells mediated by flagella [36], induction of mucus production [62], production of hemolysis and binding to ECM molecules [7], as well as toxin production [9] are examples of such characteristic of aEPEC.

## Conclusions

Taken together our results suggest that flagellin may play a role in the *in vitro* interaction of BA2103 with cellular fibronectin, and GroEL can be an accessory protein, in this process as an additional strategy acquired by this strain. Besides, considering ours and previous data, it is clear that these data cannot be extended to all O26:H11 aEPEC strains. The significance of these proteins in host cell interaction of other aEPEC serotypes is under investigation by our group.

## Availability of supporting data

All the supporting data are included as additional files.

## Additional files

Additional file 1: Figure S1. Multiple sequence alignment of flagellin from *Escherichia coli* O26:H11 strains. Sequence alignment was performed using MUSCLE server. (DOCX 22 kb) Additional file 2: Figure S2. Sequence alignment of the hypervariable region of aEPEC BA2103 flagellin and the fibronectin-binding region of *Staphylococcus aureus* FnBPA. A. Predicted amino acid sequence of aEPEC BA2103 flagellin (FliC_BA2103_), highlighting the hypervariable region (yellow). B. Amino acid sequence of *S. aureus* FnBPA, highlighting the Fn-binding region (grey). C. BLASTp results showing a 14-aa sequence of FliC_BA2103_ (residues 198 to 211 - query) with 64 % of similarity to two motifs of FnBPA (residues 764 to 777 and 802 to 815 - subject). D. Multiple sequence alignment of these three regions highlighting the conserved (dark grey) and similar (grey) amino acid residues. (DOCX 167 kb) Additional file 3: Figure S3. Amino acids sequences alignment of GroEL. The first sequence corresponding *to E. coli* J96 O4:K6 (gi|18028158), which was identified by MALDI-TOF of BA2103 isolated after fibronectin-associated immunoprecipitation. The sequence was compare to GroEL of *Shigella sonnei* 3233–85 (gi|391279593) and *E. coli* 11368 O26:H11 (gi|260853213:5396024–5397670) using BioEdit Sequence Alignment Editor vs 7.2.5.0 after alignment by ClustalW. The open box shows the amino acids differences among sequences at GroEL equatorial domain 1. The tagged sequence corresponding to conserved hydrophobic binding domain of GroEL. (DOCX 26 kb) Additional file 4: Figure S4. Flagellin purification and characterization. A. Nitrocellulose membrane containing 10 μg of purified flagellin. Immunoblotting reaction was carried out using anti-H11 rabbit serum (1) and naïve rabbit serum (2), followed by goat IgG anti-rabbit peroxidase conjugate. Arrow indicates flagellin. B. Titration of anti-H11 and naïve rabbit sera by indirect ELISA. (TIFF 26 kb)
