# Supplementary material for: Flagellin and GroEL mediates in vitro binding of an atypical enteropathogenic Escherichia coli to cellular fibronectin
Source: BMC Microbiol. 2015 Dec 18;15:278. doi: 10.1186/s12866-015-0612-4 (PMC4683701; doi:10.1186/s12866-015-0612-4)
Supplement: Additional file 1: Figure S1. — Multiple sequence alignment of flagellin from Escherichia coli O26:H11 strains. Sequence alignment was performed using MUSCLE server. (DOCX 22 kb) [file 12866_2015_612_MOESM1_ESM.docx]

**BA2103 MAQVINTNSLSLLTQNNLNKSQSSLSSAIERLSSGLRINSAKDDAAGQAIANRFTANIKG**

CFSAN001629 MAQVINTNSLSLLTQNNLNKSQSSLSSAIERLSSGLRINSAKDDAAGQAIANRFTANIKG

CVM10030 MAQVINTNSLSLLTQNNLNKSQSSLSSAIERLSSGLRINSAKDDAAGQAIANRFTANIKG

CVM10021 MAQVINTNSLSLLTQNNLNKSQSSLSSAIERLSSGLRINSAKDDAAGQAIANRFTANIKG

CVM10224 MAQVINTNSLSLLTQNNLNKSQSSLSSAIERLSSGLRINSAKDDAAGQAIANRFTANIKG

CVM9942 MAQVINTNSLSLLTQNNLNKSQSSLSSAIERLSSGLRINSAKDDAAGQAIANRFTANIKG

CVM10026 MAQVINTNSLSLLTQNNLNKSQSSLSSAIERLSSGLRINSAKDDAAGQAIANRFTANIKG

36079 MAQVINTNSLSLLTQNNLNKSQSSLSSAIERLSSGLRINSAKDDAAGQAIANRFTANIKG

36084 MAQVINTNSLSLLTQNNLNKSQSSLSSAIERLSSGLRINSAKDDAAGQAIANRFTANIKG

36943 MAQVINTNSLSLLTQNNLNKSQSSLSSAIERLSSGLRINSAKDDAAGQAIANRFTANIKG

36293 MAQVINTNSLSLLTQNNLNKSQSSLSSAIERLSSGLRINSAKDDAAGQAIANRFTANIKG

36708 MAQVINTNSLSLLTQNNLNKSQSSLSSAIERLSSGLRINSAKDDAAGQAIANRFTANIKG

34870 MAQVINTNSLSLLTQNNLNKSQSSLSSAIERLSSGLRINSAKDDAAGQAIANRFTANIKG

34827 MAQVINTNSLSLLTQNNLNKSQSSLSSAIERLSSGLRINSAKDDAAGQAIANRFTANIKG

2011C-3274 MAQVINTNSLSLLTQNNLNKSQSSLSSAIERLSSGLRINSAKDDAAGQAIANRFTANIKG

2010C-4244 MAQVINTNSLSLLTQNNLNKSQSSLSSAIERLSSGLRINSAKDDAAGQAIANRFTANIKG

2010C-3902 MAQVINTNSLSLLTQNNLNKSQSSLSSAIERLSSGLRINSAKDDAAGQAIANRFTANIKG

2010C-3871 MAQVINTNSLSLLTQNNLNKSQSSLSSAIERLSSGLRINSAKDDAAGQAIANRFTANIKG

2010C-3472 MAQVINTNSLSLLTQNNLNKSQSSLSSAIERLSSGLRINSAKDDAAGQAIANRFTANIKG

2010C-3051 MAQVINTNSLSLLTQNNLNKSQSSLSSAIERLSSGLRINSAKDDAAGQAIANRFTANIKG

2009C-4826 MAQVINTNSLSLLTQNNLNKSQSSLSSAIERLSSGLRINSAKDDAAGQAIANRFTANIKG

2009C-4760 MAQVINTNSLSLLTQNNLNKSQSSLSSAIERLSSGLRINSAKDDAAGQAIANRFTANIKG

2009C-3996 MAQVINTNSLSLLTQNNLNKSQSSLSSAIERLSSGLRINSAKDDAAGQAIANRFTANIKG

2009C-3612 MAQVINTNSLSLLTQNNLNKSQSSLSSAIERLSSGLRINSAKDDAAGQAIANRFTANIKG

2009C-3689 MAQVINTNSLSLLTQNNLNKSQSSLSSAIERLSSGLRINSAKDDAAGQAIANRFTANIKG

2011C-3655 MAQVINTNSLSLLTQNNLNKSQSSLSSAIERLSSGLRINSAKDDAAGQAIANRFTANIKG

2011C-3282 MAQVINTNSLSLLTQNNLNKSQSSLSSAIERLSSGLRINSAKDDAAGQAIANRFTANIKG

2011C-3506 MAQVINTNSLSLLTQNNLNKSQSSLSSAIERLSSGLRINSAKDDAAGQAIANRFTANIKG

2011C-3387 MAQVINTNSLSLLTQNNLNKSQSSLSSAIERLSSGLRINSAKDDAAGQAIANRFTANIKG

2011C-3270 MAQVINTNSLSLLTQNNLNKSQSSLSSAIERLSSGLRINSAKDDAAGQAIANRFTANIKG

2010EL-1699 MAQVINTNSLSLLTQNNLNKSQSSLSSAIERLSSGLRINSAKDDAAGQAIANRFTANIKG

2010C-5028 MAQVINTNSLSLLTQNNLNKSQSSLSSAIERLSSGLRINSAKDDAAGQAIANRFTANIKG

2010C-4834 MAQVINTNSLSLLTQNNLNKSQSSLSSAIERLSSGLRINSAKDDAAGQAIANRFTANIKG

2010C-4819 MAQVINTNSLSLLTQNNLNKSQSSLSSAIERLSSGLRINSAKDDAAGQAIANRFTANIKG

2010C-4430 MAQVINTNSLSLLTQNNLNKSQSSLSSAIERLSSGLRINSAKDDAAGQAIANRFTANIKG

03-3500 MAQVINTNSLSLLTQNNLNKSQSSLSSAIERLSSGLRINSAKDDAAGQAIANRFTANIKG

06-3464 MAQVINTNSLSLLTQNNLNKSQSSLSSAIERLSSGLRINSAKDDAAGQAIANRFTANIKG

05-3646 MAQVINTNSLSLLTQNNLNKSQSSLSSAIERLSSGLRINSAKDDAAGQAIANRFTANIKG

11368 MAQVINTNSLSLLTQNNLNKSQSSLSSAIERLSSGLRINSAKDDAAGQAIANRFTANIKG

97-3250 MAQVINTNSLSLLTQNNLNKSQSSLSSAIERLSSGLRINSAKDDAAGQAIANRFTANIKG

DEC10D MAQVINTNSLSLLTQNNLNKSQSSLSSAIERLSSGLRINSAKDDAAGQAIANRFTANIKG

DEC10C MAQVINTNSLSLLTQNNLNKSQSSLSSAIERLSSGLRINSAKDDAAGQAIANRFTANIKG

DEC10B MAQVINTNSLSLLTQNNLNKSQSSLSSAIERLSSGLRINSAKDDAAGQAIANRFTANIKG

DEC9E MAQVINTNSLSLLTQNNLNKSQSSLSSAIERLSSGLRINSAKDDAAGQAIANRFTANIKG

DEC9D MAQVINTNSLSLLTQNNLNKSQSSLSSAIERLSSGLRINSAKDDAAGQAIANRFTANIKG

DEC9A MAQVINTNSLSLLTQNNLNKSQSSLSSAIERLSSGLRINSAKDDAAGQAIANRFTANIKG

36348 MAQVINTNSLSLLTQNNLNKSQSSLSSAIERLSSGLRINSAKDDAAGQAIANRFTANIKG

************************************************************

**BA2103 LTQASRNANDGISVAQTTEGALNEINNNLQRVRELTVQATNGTNSDSDLSSIQAEITQRL**

CFSAN001629 LTQASRNANDGISVAQTTEGALNEINNNLQRVRELTVQATNGTNSDSDLSSIQAEITQRL

CVM10030 LTQASRNANDGISVAQTTEGALNEINNNLQRVRELTVQATNGTNSDSDLSSIQAEITQRL

CVM10021 LTQASRNANDGISVAQTTEGALNEINNNLQRVRELTVQATNGTNSDSDLSSIQAEITQRL

CVM10224 LTQASRNANDGISVAQTTEGALNEINNNLQRVRELTVQATNGTNSDSDLSSIQAEITQRL

CVM9942 LTQASRNANDGISVAQTTEGALNEINNNLQRVRELTVQATNGTNSDSDLSSIQAEITQRL

CVM10026 LTQASRNANDGISVAQTTEGALNEINNNLQRVRELTVQATNGTNSDSDLSSIQAEITQRL

36079 LTQASRNANDGISVAQTTEGALNEINNNLQRVRELTVQATNGTNSDSDLSSIQAEITQRL

36084 LTQASRNANDGISVAQTTEGALNEINNNLQRVRELTVQATNGTNSDSDLSSIQAEITQRL

36943 LTQASRNANDGISVAQTTEGALNEINNNLQRVRELTVQATNGTNSDSDLSSIQAEITQRL

36293 LTQASRNANDGISVAQTTEGALNEINNNLQRVRELTVQATNGTNSDSDLSSIQAEITQRL

36708 LTQASRNANDGISVAQTTEGALNEINNNLQRVRELTVQATNGTNSDSDLSSIQAEITQRL

34870 LTQASRNANDGISVAQTTEGALNEINNNLQRVRELTVQATNGTNSDSDLSSIQAEITQRL

34827 LTQASRNANDGISVAQTTEGALNEINNNLQRVRELTVQATNGTNSDSDLSSIQAEITQRL

2011C-3274 LTQASRNANDGISVAQTTEGALNEINNNLQRVRELTVQATNGTNSDSDLSSIQAEITQRL

2010C-4244 LTQASRNANDGISVAQTTEGALNEINNNLQRVRELTVQATNGTNSDSDLSSIQAEITQRL

2010C-3902 LTQASRNANDGISVAQTTEGALNEINNNLQRVRELTVQATNGTNSDSDLSSIQAEITQRL

2010C-3871 LTQASRNANDGISVAQTTEGALNEINNNLQRVRELTVQATNGTNSDSDLSSIQAEITQRL

2010C-3472 LTQASRNANDGISVAQTTEGALNEINNNLQRVRELTVQATNGTNSDSDLSSIQAEITQRL

2010C-3051 LTQASRNANDGISVAQTTEGALNEINNNLQRVRELTVQATNGTNSDSDLSSIQAEITQRL

2009C-4826 LTQASRNANDGISVAQTTEGALNEINNNLQRVRELTVQATNGTNSDSDLSSIQAEITQRL

2009C-4760 LTQASRNANDGISVAQTTEGALNEINNNLQRVRELTVQATNGTNSDSDLSSIQAEITQRL

2009C-3996 LTQASRNANDGISVAQTTEGALNEINNNLQRVRELTVQATNGTNSDSDLSSIQAEITQRL

2009C-3612 LTQASRNANDGISVAQTTEGALNEINNNLQRVRELTVQATNGTNSDSDLSSIQAEITQRL

2009C-3689 LTQASRNANDGISVAQTTEGALNEINNNLQRVRELTVQATNGTNSDSDLSSIQAEITQRL

2011C-3655 LTQASRNANDGISVAQTTEGALNEINNNLQRVRELTVQATNGTNSDSDLSSIQAEITQRL

2011C-3282 LTQASRNANDGISVAQTTEGALNEINNNLQRVRELTVQATNGTNSDSDLSSIQAEITQRL

2011C-3506 LTQASRNANDGISVAQTTEGALNEINNNLQRVRELTVQATNGTNSDSDLSSIQAEITQRL

2011C-3387 LTQASRNANDGISVAQTTEGALNEINNNLQRVRELTVQATNGTNSDSDLSSIQAEITQRL

2011C-3270 LTQASRNANDGISVAQTTEGALNEINNNLQRVRELTVQATNGTNSDSDLSSIQAEITQRL

2010EL-1699 LTQASRNANDGISVAQTTEGALNEINNNLQRVRELTVQATNGTNSDSDLSSIQAEITQRL

2010C-5028 LTQASRNANDGISVAQTTEGALNEINNNLQRVRELTVQATNGTNSDSDLSSIQAEITQRL

2010C-4834 LTQASRNANDGISVAQTTEGALNEINNNLQRVRELTVQATNGTNSDSDLSSIQAEITQRL

2010C-4819 LTQASRNANDGISVAQTTEGALNEINNNLQRVRELTVQATNGTNSDSDLSSIQAEITQRL

2010C-4430 LTQASRNANDGISVAQTTEGALNEINNNLQRVRELTVQATNGTNSDSDLSSIQAEITQRL

03-3500 LTQASRNANDGISVAQTTEGALNEINNNLQRVRELTVQATNGTNSDSDLSSIQAEITQRL

06-3464 LTQASRNANDGISVAQTTEGALNEINNNLQRVRELTVQATNGTNSDSDLSSIQAEITQRL

05-3646 LTQASRNANDGISVAQTTEGALNEINNNLQRVRELTVQATNGTNSDSDLSSIQAEITQRL

11368 LTQASRNANDGISVAQTTEGALNEINNNLQRVRELTVQATNGTNSDSDLSSIQAEITQRL

97-3250 LTQASRNANDGISVAQTTEGALNEINNNLQRVRELTVQATNGTNSDSDLSSIQAEITQRL

DEC10D LTQASRNANDGISVAQTTEGALNEINNNLQRVRELTVQATNGTNSDSDLSSIQAEITQRL

DEC10C LTQASRNANDGISVAQTTEGALNEINNNLQRVRELTVQATNGTNSDSDLSSIQAEITQRL

DEC10B LTQASRNANDGISVAQTTEGALNEINNNLQRVRELTVQATNGTNSDSDLSSIQAEITQRL

DEC9E LTQASRNANDGISVAQTTEGALNEINNNLQRVRELTVQATNGTNSDSDLSSIQAEITQRL

DEC9D LTQASRNANDGISVAQTTEGALNEINNNLQRVRELTVQATNGTNSDSDLSSIQAEITQRL

DEC9A LTQASRNANDGISVAQTTEGALNEINNNLQRVRELTVQATNGTNSDSDLSSIQAEITQRL

36348 LTQASRNANDGISVAQTTEGALNEINNNLQRVRELTVQATNGTNTDSDLSSIQAEITQRL

********************************************:***************

**BA2103 EEIDRVSEQTQFNGVKVLAENNEMKIQVGANDGETITINLAKIDAKTLGLDGFNIDGAQK**

CFSAN001629 EEIDRVSEQTQFNGVKVLAENNEMKIQVGANDGETITINLAKIDAKTLGLDGFNIDGAQK

CVM10030 EEIDRVSEQTQFNGVKVLAENNEMKIQVGANDGETITINLAKIDAKTLGLDGFNIDGAQK

CVM10021 EEIDRVSEQTQFNGVKVLAENNEMKIQVGANDGETITINLAKIDAKTLGLDGFNIDGAQK

CVM10224 EEIDRVSEQTQFNGVKVLAENNEMKIQVGANDGETITINLAKIDAKTLGLDGFNIDGAQK

CVM9942 EEIDRVSEQTQFNGVKVLAENNEMKIQVGANDGETITINLAKIDAKTLGLDGFNIDGAQK

CVM10026 EEIDRVSEQTQFNGVKVLAENNEMKIQVGANDGETITINLAKIDAKTLGLDGFNIDGAQK

36079 EEIDRVSEQTQFNGVKVLAENNEMKIQVGANDGETITINLAKIDAKTLGLDGFNIDGAQK

36084 EEIDRVSEQTQFNGVKVLAENNEMKIQVGANDGETITINLAKIDAKTLGLDGFNIDGAQK

36943 EEIDRVSEQTQFNGVKVLAENNEMKIQVGANDGETITINLAKIDAKTLGLDGFNIDGAQK

36293 EEIDRVSEQTQFNGVKVLAENNEMKIQVGANDGETITINLAKIDAKTLGLDGFNIDGAQK

36708 EEIDRVSEQTQFNGVKVLAENNEMKIQVGANDGETITINLAKIDAKTLGLDGFNIDGAQK

34870 EEIDRVSEQTQFNGVKVLAENNEMKIQVGANDGETITINLAKIDAKTLGLDGFNIDGAQK

34827 EEIDRVSEQTQFNGVKVLAENNEMKIQVGANDGETITINLAKIDAKTLGLDGFNIDGAQK

2011C-3274 EEIDRVSEQTQFNGVKVLAENNEMKIQVGANDGETITINLAKIDAKTLGLDGFNIDGAQK

2010C-4244 EEIDRVSEQTQFNGVKVLAENNEMKIQVGANDGETITINLAKIDAKTLGLDGFNIDGAQK

2010C-3902 EEIDRVSEQTQFNGVKVLAENNEMKIQVGANDGETITINLAKIDAKTLGLDGFNIDGAQK

2010C-3871 EEIDRVSEQTQFNGVKVLAENNEMKIQVGANDGETITINLAKIDAKTLGLDGFNIDGAQK

2010C-3472 EEIDRVSEQTQFNGVKVLAENNEMKIQVGANDGETITINLAKIDAKTLGLDGFNIDGAQK

2010C-3051 EEIDRVSEQTQFNGVKVLAENNEMKIQVGANDGETITINLAKIDAKTLGLDGFNIDGAQK

2009C-4826 EEIDRVSEQTQFNGVKVLAENNEMKIQVGANDGETITINLAKIDAKTLGLDGFNIDGAQK

2009C-4760 EEIDRVSEQTQFNGVKVLAENNEMKIQVGANDGETITINLAKIDAKTLGLDGFNIDGAQK

2009C-3996 EEIDRVSEQTQFNGVKVLAENNEMKIQVGANDGETITINLAKIDAKTLGLDGFNIDGAQK

2009C-3612 EEIDRVSEQTQFNGVKVLAENNEMKIQVGANDGETITINLAKIDAKTLGLDGFNIDGAQK

2009C-3689 EEIDRVSEQTQFNGVKVLAENNEMKIQVGANDGETITINLAKIDAKTLGLDGFNIDGAQK

2011C-3655 EEIDRVSEQTQFNGVKVLAENNEMKIQVGANDGETITINLAKIDAKTLGLDGFNIDGAQK

2011C-3282 EEIDRVSEQTQFNGVKVLAENNEMKIQVGANDGETITINLAKIDAKTLGLDGFNIDGAQK

2011C-3506 EEIDRVSEQTQFNGVKVLAENNEMKIQVGANDGETITINLAKIDAKTLGLDGFNIDGAQK

2011C-3387 EEIDRVSEQTQFNGVKVLAENNEMKIQVGANDGETITINLAKIDAKTLGLDGFNIDGAQK

2011C-3270 EEIDRVSEQTQFNGVKVLAENNEMKIQVGANDGETITINLAKIDAKTLGLDGFNIDGAQK

2010EL-1699 EEIDRVSEQTQFNGVKVLAENNEMKIQVGANDGETITINLAKIDAKTLGLDGFNIDGAQK

2010C-5028 EEIDRVSEQTQFNGVKVLAENNEMKIQVGANDGETITINLAKIDAKTLGLDGFNIDGAQK

2010C-4834 EEIDRVSEQTQFNGVKVLAENNEMKIQVGANDGETITINLAKIDAKTLGLDGFNIDGAQK

2010C-4819 EEIDRVSEQTQFNGVKVLAENNEMKIQVGANDGETITINLAKIDAKTLGLDGFNIDGAQK

2010C-4430 EEIDRVSEQTQFNGVKVLAENNEMKIQVGANDGETITINLAKIDAKTLGLDGFNIDGAQK

03-3500 EEIDRVSEQTQFNGVKVLAENNEMKIQVGANDGETITINLAKIDAKTLGLDGFNIDGAQK

06-3464 EEIDRVSEQTQFNGVKVLAENNEMKIQVGANDGETITINLAKIDAKTLGLDGFNIDGAQK

05-3646 EEIDRVSEQTQFNGVKVLAENNEMKIQVGANDGETITINLAKIDAKTLGLDGFNIDGAQK

11368 EEIDRVSEQTQFNGVKVLAENNEMKIQVGANDGETITINLAKIDAKTLGLDGFNIDGAQK

97-3250 EEIDRVSEQTQFNGVKVLAENNEMKIQVGANDGETITINLAKIDAKTLGLDGFNIDGAQK

DEC10D EEIDRVSEQTQFNGVKVLAENNEMKIQVGANDGETITINLAKIDAKTLGLDGFNIDGAQK

DEC10C EEIDRVSEQTQFNGVKVLAENNEMKIQVGANDGETITINLAKIDAKTLGLDGFNIDGAQK

DEC10B EEIDRVSEQTQFNGVKVLAENNEMKIQVGANDGETITINLAKIDAKTLGLDGFNIDGAQK

DEC9E EEIDRVSEQTQFNGVKVLAENNEMKIQVGANDGETITINLAKIDAKTLGLDGFNIDGAQK

DEC9D EEIDRVSEQTQFNGVKVLAENNEMKIQVGANDGETITINLAKIDAKTLGLDGFNIDGAQK

DEC9A EEIDRVSEQTQFNGVKVLAENNEMKIQVGANDGETITINLAKIDAKTLGLDGFNIDGAQK

36348 EEIDRVSEQTQFNGVKVLAENNEMKIQVGANDGETITINLAKIDAKTLGLDGFNIDGAQK

************************************************************

**BA2103 ATGSDLISKFKATGTDNYDVGGDAYTVNVDSGAVKDTTGNDIFVSAADGSLTTKSDTNIA**

CFSAN001629 ATGSDLISKFKATGTDNYDVGGDAYTVNVDSGAVKDTTGNDIFVSAADGSLTTKSDTNIA

CVM10030 ATGSDLISKFKATGTDNYDVGGDAYTVNVDSGAVKDTTGNDIFVSAADGSLTTKSDTNIA

CVM10021 ATGSDLISKFKATGTDNYDVGGDAYTVNVDSGAVKDTTGNDIFVSAADGSLTTKSDTNIA

CVM10224 ATGSDLISKFKATGTDNYDVGGDAYTVNVDSGAVKDTTGNDIFVSAADGSLTTKSDTNIA

CVM9942 ATGSDLISKFKATGTDNYDVGGDAYTVNVDSGAVKDTTGNDIFVSAADGSLTTKSDTNIA

CVM10026 ATGSDLISKFKATGTDNYDVGGDAYTVNVDSGAVKDTTGNDIFVSAADGSLTTKSDTNIA

36079 ATGSDLISKFKATGTDNYDVGGDAYTVNVDSGAVKDTTGNDIFVSAADGSLTTKSDTNIA

36084 ATGSDLISKFKATGTDNYDVGGDAYTVNVDSGAVKDTTGNDIFVSAADGSLTTKSDTNIA

36943 ATGSDLISKFKATGTDNYDVGGDAYTVNVDSGAVKDTTGNDIFVSAADGSLTTKSDTNIA

36293 ATGSDLISKFKATGTDNYDVGGDAYTVNVDSGAVKDTTGNDIFVSAADGSLTTKSDTNIA

36708 ATGSDLISKFKATGTDNYDVGGDAYTVNVDSGAVKDTTGNDIFVSAADGSLTTKSDTNIA

34870 ATGSDLISKFKATGTDNYDVGGDAYTVNVDSGAVKDTTGNDIFVSAADGSLTTKSDTNIA

34827 ATGSDLISKFKATGTDNYDVGGDAYTVNVDSGAVKDTTGNDIFVSAADGSLTTKSDTNIA

2011C-3274 ATGSDLISKFKATGTDNYDVGGDAYTVNVDSGAVKDTTGNDIFVSAADGSLTTKSDTNIA

2010C-4244 ATGSDLISKFKATGTDNYDVGGDAYTVNVDSGAVKDTTGNDIFVSAADGSLTTKSDTNIA

2010C-3902 ATGSDLISKFKATGTDNYDVGGDAYTVNVDSGAVKDTTGNDIFVSAADGSLTTKSDTNIA

2010C-3871 ATGSDLISKFKATGTDNYDVGGDAYTVNVDSGAVKDTTGNDIFVSAADGSLTTKSDTNIA

2010C-3472 ATGSDLISKFKATGTDNYDVGGDAYTVNVDSGAVKDTTGNDIFVSAADGSLTTKSDTNIA

2010C-3051 ATGSDLISKFKATGTDNYDVGGDAYTVNVDSGAVKDTTGNDIFVSAADGSLTTKSDTNIA

2009C-4826 ATGSDLISKFKATGTDNYDVGGDAYTVNVDSGAVKDTTGNDIFVSAADGSLTTKSDTNIA

2009C-4760 ATGSDLISKFKATGTDNYDVGGDAYTVNVDSGAVKDTTGNDIFVSAADGSLTTKSDTNIA

2009C-3996 ATGSDLISKFKATGTDNYDVGGDAYTVNVDSGAVKDTTGNDIFVSAADGSLTTKSDTNIA

2009C-3612 ATGSDLISKFKATGTDNYDVGGDAYTVNVDSGAVKDTTGNDIFVSAADGSLTTKSDTNIA

2009C-3689 ATGSDLISKFKATGTDNYDVGGDAYTVNVDSGAVKDTTGNDIFVSAADGSLTTKSDTNIA

2011C-3655 ATGSDLISKFKATGTDNYDVGGDAYTVNVDSGAVKDTTGNDIFVSAADGSLTTKSDTNIA

2011C-3282 ATGSDLISKFKATGTDNYDVGGDAYTVNVDSGAVKDTTGNDIFVSAADGSLTTKSDTNIA

2011C-3506 ATGSDLISKFKATGTDNYDVGGDAYTVNVDSGAVKDTTGNDIFVSAADGSLTTKSDTNIA

2011C-3387 ATGSDLISKFKATGTDNYDVGGDAYTVNVDSGAVKDTTGNDIFVSAADGSLTTKSDTNIA

2011C-3270 ATGSDLISKFKATGTDNYDVGGDAYTVNVDSGAVKDTTGNDIFVSAADGSLTTKSDTNIA

2010EL-1699 ATGSDLISKFKATGTDNYDVGGDAYTVNVDSGAVKDTTGNDIFVSAADGSLTTKSDTNIA

2010C-5028 ATGSDLISKFKATGTDNYDVGGDAYTVNVDSGAVKDTTGNDIFVSAADGSLTTKSDTNIA

2010C-4834 ATGSDLISKFKATGTDNYDVGGDAYTVNVDSGAVKDTTGNDIFVSAADGSLTTKSDTNIA

2010C-4819 ATGSDLISKFKATGTDNYDVGGDAYTVNVDSGAVKDTTGNDIFVSAADGSLTTKSDTNIA

2010C-4430 ATGSDLISKFKATGTDNYDVGGDAYTVNVDSGAVKDTTGNDIFVSAADGSLTTKSDTNIA

03-3500 ATGSDLISKFKATGTDNYDVGGDAYTVNVDSGAVKDTTGNDIFVSAADGSLTTKSDTNIA

06-3464 ATGSDLISKFKATGTDNYDVGGDAYTVNVDSGAVKDTTGNDIFVSAADGSLTTKSDTNIA

05-3646 ATGSDLISKFKATGTDNYDVGGDAYTVNVDSGAVKDTTGNDIFVSAADGSLTTKSDTNIA

11368 ATGSDLISKFKATGTDNYDVGGDAYTVNVDSGAVKDTTGNDIFVSAADGSLTTKSDTNIA

97-3250 ATGSDLISKFKATGTDNYDVGGDAYTVNVDSGAVKDTTGNDIFVSAADGSLTTKSDTNIA

DEC10D ATGSDLISKFKATGTDNYDVGGDAYTVNVDSGAVKDTTGNDIFVSAADGSLTTKSDTNIA

DEC10C ATGSDLISKFKATGTDNYDVGGDAYTVNVDSGAVKDTTGNDIFVSAADGSLTTKSDTNIA

DEC10B ATGSDLISKFKATGTDNYDVGGDAYTVNVDSGAVKDTTGNDIFVSAADGSLTTKSDTNIA

DEC9E ATGSDLISKFKATGTDNYDVGGDAYTVNVDSGAVKDTTGNDIFVSAADGSLTTKSDTNIA

DEC9D ATGSDLISKFKATGTDNYDVGGDAYTVNVDSGAVKDTTGNDIFVSAADGSLTTKSDTNIA

DEC9A ATGSDLISKFKATGTDNYDVGGDAYTVNVDSGAVKDTTGNDIFVSAADGSLTTKSDTNIA

36348 ATGSDLISKFKATGTDNYDVGGDAYTVNVDSGAVKDTTGNDIFVSAADGSLTTKSDTNIA

************************************************************

**BA2103 GTGIDATALAAAAKNKAQNDKFTFNGVEFTTTTAADGNGNGVYSAEIDGKSVTFTVTDAD**

CFSAN001629 GTGIDATALAAAAKNKAQNDKFTFNGVEFTTTTAADGNGNGVYSAEIDGKSVTFTVTDAD

CVM10030 GTGIDATALAAAAKNKAQNDKFTFNGVEFTTTTAADGNGNGVYSAEIDGKSVTFTVTDAD

CVM10021 GTGIDATALAAAAKNKAQNDKFTFNGVEFTTTTAADGNGNGVYSAEIDGKSVTFTVTDAD

CVM10224 GTGIDATALAAAAKNKAQNDKFTFNGVEFTTTTAADGNGNGVYSAEIDGKSVTFTVTDAD

CVM9942 GTGIDATALAAAAKNKAQNDKFTFNGVEFTTTTAADGNGNGVYSAEIDGKSVTFTVTDAD

CVM10026 GTGIDATALAAAAKNKAQNDKFTFNGVEFTTTTAADGNGNGVYSAEIDGKSVTFTVTDAD

36079 GTGIDATALAAAAKNKAQNDKFTFNGVEFTTTTAADGNGNGVYSAEIDGKSVTFTVTDAD

36084 GTGIDATALAAAAKNKAQNDKFTFNGVEFTTTTAADGNGNGVYSAEIDGKSVTFTVTDAD

36943 GTGIDATALAAAAKNKAQNDKFTFNGVEFTTTTAADGNGNGVYSAEIDGKSVTFTVTDAD

36293 GTGIDATALAAAAKNKAQNDKFTFNGVEFTTTTAADGNGNGVYSAEIDGKSVTFTVTDAD

36708 GTGIDATALAAAAKNKAQNDKFTFNGVEFTTTTAADGNGNGVYSAEIDGKSVTFTVTDAD

34870 GTGIDATALAAAAKNKAQNDKFTFNGVEFTTTTAADGNGNGVYSAEIDGKSVTFTVTDAD

34827 GTGIDATALAAAAKNKAQNDKFTFNGVEFTTTTAADGNGNGVYSAEIDGKSVTFTVTDAD

2011C-3274 GTGIDATALAAAAKNKAQNDKFTFNGVEFTTTTAADGNGNGVYSAEIDGKSVTFTVTDAD

2010C-4244 GTGIDATALAAAAKNKAQNDKFTFNGVEFTTTTAADGNGNGVYSAEIDGKSVTFTVTDAD

2010C-3902 GTGIDATALAAAAKNKAQNDKFTFNGVEFTTTTAADGNGNGVYSAEIDGKSVTFTVTDAD

2010C-3871 GTGIDATALAAAAKNKAQNDKFTFNGVEFTTTTAADGNGNGVYSAEIDGKSVTFTVTDAD

2010C-3472 GTGIDATALAAAAKNKAQNDKFTFNGVEFTTTTAADGNGNGVYSAEIDGKSVTFTVTDAD

2010C-3051 GTGIDATALAAAAKNKAQNDKFTFNGVEFTTTTAADGNGNGVYSAEIDGKSVTFTVTDAD

2009C-4826 GTGIDATALAAAAKNKAQNDKFTFNGVEFTTTTAADGNGNGVYSAEIDGKSVTFTVTDAD

2009C-4760 GTGIDATALAAAAKNKAQNDKFTFNGVEFTTTTAADGNGNGVYSAEIDGKSVTFTVTDAD

2009C-3996 GTGIDATALAAAAKNKAQNDKFTFNGVEFTTTTAADGNGNGVYSAEIDGKSVTFTVTDAD

2009C-3612 GTGIDATALAAAAKNKAQNDKFTFNGVEFTTTTAADGNGNGVYSAEIDGKSVTFTVTDAD

2009C-3689 GTGIDATALAAAAKNKAQNDKFTFNGVEFTTTTAADGNGNGVYSAEIDGKSVTFTVTDAD

2011C-3655 GTGIDATALAAAAKNKAQNDKFTFNGVEFTTTTAADGNGNGVYSAEIDGKSVTFTVTDAD

2011C-3282 GTGIDATALAAAAKNKAQNDKFTFNGVEFTTTTAADGNGNGVYSAEIDGKSVTFTVTDAD

2011C-3506 GTGIDATALAAAAKNKAQNDKFTFNGVEFTTTTAADGNGNGVYSAEIDGKSVTFTVTDAD

2011C-3387 GTGIDATALAAAAKNKAQNDKFTFNGVEFTTTTAADGNGNGVYSAEIDGKSVTFTVTDAD

2011C-3270 GTGIDATALAAAAKNKAQNDKFTFNGVEFTTTTAADGNGNGVYSAEIDGKSVTFTVTDAD

2010EL-1699 GTGIDATALAAAAKNKAQNDKFTFNGVEFTTTTAADGNGNGVYSAEIDGKSVTFTVTDAD

2010C-5028 GTGIDATALAAAAKNKAQNDKFTFNGVEFTTTTAADGNGNGVYSAEIDGKSVTFTVTDAD

2010C-4834 GTGIDATALAAAAKNKAQNDKFTFNGVEFTTTTAADGNGNGVYSAEIDGKSVTFTVTDAD

2010C-4819 GTGIDATALAAAAKNKAQNDKFTFNGVEFTTTTAADGNGNGVYSAEIDGKSVTFTVTDAD

2010C-4430 GTGIDATALAAAAKNKAQNDKFTFNGVEFTTTTAADGNGNGVYSAEIDGKSVTFTVTDAD

03-3500 GTGIDATALAAAAKNKAQNDKFTFNGVEFTTTTAADGNGNGVYSAEIDGKSVTFTVTDAD

06-3464 GTGIDATALAAAAKNKAQNDKFTFNGVEFTTTTAADGNGNGVYSAEIDGKSVTFTVTDAD

05-3646 GTGIDATALAAAAKNKAQNDKFTFNGVEFTTTTAADGNGNGVYSAEIDGKSVTFTVTDAD

11368 GTGIDATALAAAAKNKAQNDKFTFNGVEFTTTTAADGNGNGVYSAEIDGKSVTFTVTDAD

97-3250 GTGIDATALAAAAKNKAQNDKFTFNGVEFTTTTAADGNGNGVYSAEIDGKSVTFTVTDAD

DEC10D GTGIDATALAAAAKNKAQNDKFTFNGVEFTTTTAADGNGNGVYSAEIDGKSVTFTVTDAD

DEC10C GTGIDATALAAAAKNKAQNDKFTFNGVEFTTTTAADGNGNGVYSAEIDGKSVTFTVTDAD

DEC10B GTGIDATALAAAAKNKAQNDKFTFNGVEFTTTTAADGNGNGVYSAEIDGKSVTFTVTDAD

DEC9E GTGIDATALAAAAKNKAQNDKFTFNGVEFTTTTAADGNGNGVYSAEIDGKSVTFTVTDAD

DEC9D GTGIDATALAAAAKNKAQNDKFTFNGVEFTTTTAADGNGNGVYSAEIDGKSVTFTVTDAD

DEC9A GTGIDATALAAAAKNKAQNDKFTFNGVEFTTTTAADGNGNGVYSAEIDGKSVTFTVTDAD

36348 GTGIDATALAAAAKNKAQNDKFTFNGVEFTTTTAADGNGNGVYSAEIDGKSVTFTVTDAD

************************************************************

**BA2103 KKASLITSETVYKNSAGLYTTTKVDNKAATLSDLDLNAAKKTGSTLVVNGATYDVSADGK**

CFSAN001629 KKASLITSETVYKNSAGLYTTTKVDNKAATLSDLDLNAAKKTGSTLVVNGATYDVSADGK

CVM10030 KKASLITSETVYKNSAGLYTTTKVDNKAATLSDLDLNAAKKTGSTLVVNGATYDVSADGK

CVM10021 KKASLITSETVYKNSAGLYTTTKVDNKAATLSDLDLNAAKKTGSTLVVNGATYDVSADGK

CVM10224 KKASLITSETVYKNSAGLYTTTKVDNKAATLSDLDLNAAKKTGSTLVVNGATYDVSADGK

CVM9942 KKASLITSETVYKNSAGLYTTTKVDNKAATLSDLDLNAAKKTGSTLVVNGATYDVSADGK

CVM10026 KKASLITSETVYKNSAGLYTTTKVDNKAATLSDLDLNAAKKTGSTLVVNGATYDVSADGK

36079 KKASLITSETVYKNSAGLYTTTKVDNKAATLSDLDLNAAKKTGSTLVVNGATYDVSADGK

36084 KKASLITSETVYKNSAGLYTTTKVDNKAATLSDLDLNAAKKTGSTLVVNGATYDVSADGK

36943 KKASLITSETVYKNSAGLYTTTKVDNKAATLSDLDLNAAKKTGSTLVVNGATYDVSADGK

36293 KKASLITSETVYKNSAGLYTTTKVDNKAATLSDLDLNAAKKTGSTLVVNGATYDVSADGK

36708 KKASLITSETVYKNSAGLYTTTKVDNKAATLSDLDLNAAKKTGSTLVVNGATYDVSADGK

34870 KKASLITSETVYKNSAGLYTTTKVDNKAATLSDLDLNAAKKTGSTLVVNGATYDVSADGK

34827 KKASLITSETVYKNSAGLYTTTKVDNKAATLSDLDLNAAKKTGSTLVVNGATYDVSADGK

2011C-3274 KKASLITSETVYKNSAGLYTTTKVDNKAATLSDLDLNAAKKTGSTLVVNGATYDVSADGK

2010C-4244 KKASLITSETVYKNSAGLYTTTKVDNKAATLSDLDLNAAKKTGSTLVVNGATYDVSADGK

2010C-3902 KKASLITSETVYKNSAGLYTTTKVDNKAATLSDLDLNAAKKTGSTLVVNGATYDVSADGK

2010C-3871 KKASLITSETVYKNSAGLYTTTKVDNKAATLSDLDLNAAKKTGSTLVVNGATYDVSADGK

2010C-3472 KKASLITSETVYKNSAGLYTTTKVDNKAATLSDLDLNAAKKTGSTLVVNGATYDVSADGK

2010C-3051 KKASLITSETVYKNSAGLYTTTKVDNKAATLSDLDLNAAKKTGSTLVVNGATYDVSADGK

2009C-4826 KKASLITSETVYKNSAGLYTTTKVDNKAATLSDLDLNAAKKTGSTLVVNGATYDVSADGK

2009C-4760 KKASLITSETVYKNSAGLYTTTKVDNKAATLSDLDLNAAKKTGSTLVVNGATYDVSADGK

2009C-3996 KKASLITSETVYKNSAGLYTTTKVDNKAATLSDLDLNAAKKTGSTLVVNGATYDVSADGK

2009C-3612 KKASLITSETVYKNSAGLYTTTKVDNKAATLSDLDLNAAKKTGSTLVVNGATYDVSADGK

2009C-3689 KKASLITSETVYKNSAGLYTTTKVDNKAATLSDLDLNAAKKTGSTLVVNGATYDVSADGK

2011C-3655 KKASLITSETVYKNSAGLYTTTKVDNKAATLSDLDLNAAKKTGSTLVVNGATYDVSADGK

2011C-3282 KKASLITSETVYKNSAGLYTTTKVDNKAATLSDLDLNAAKKTGSTLVVNGATYDVSADGK

2011C-3506 KKASLITSETVYKNSAGLYTTTKVDNKAATLSDLDLNAAKKTGSTLVVNGATYDVSADGK

2011C-3387 KKASLITSETVYKNSAGLYTTTKVDNKAATLSDLDLNAAKKTGSTLVVNGATYDVSADGK

2011C-3270 KKASLITSETVYKNSAGLYTTTKVDNKAATLSDLDLNAAKKTGSTLVVNGATYDVSADGK

2010EL-1699 KKASLITSETVYKNSAGLYTTTKVDNKAATLSDLDLNAAKKTGSTLVVNGATYDVSADGK

2010C-5028 KKASLITSETVYKNSAGLYTTTKVDNKAATLSDLDLNAAKKTGSTLVVNGATYDVSADGK

2010C-4834 KKASLITSETVYKNSAGLYTTTKVDNKAATLSDLDLNAAKKTGSTLVVNGATYDVSADGK

2010C-4819 KKASLITSETVYKNSAGLYTTTKVDNKAATLSDLDLNAAKKTGSTLVVNGATYDVSADGK

2010C-4430 KKASLITSETVYKNSAGLYTTTKVDNKAATLSDLDLNAAKKTGSTLVVNGATYDVSADGK

03-3500 KKASLITSETVYKNSAGLYTTTKVDNKAATLSDLDLNAAKKTGSTLVVNGATYDVSADGK

06-3464 KKASLITSETVYKNSAGLYTTTKVDNKAATLSDLDLNAAKKTGSTLVVNGATYDVSADGK

05-3646 KKASLITSETVYKNSAGLYTTTKVDNKAATLSDLDLNAAKKTGSTLVVNGATYDVSADGK

11368 KKASLITSETVYKNSAGLYTTTKVDNKAATLSDLDLNAAKKTGSTLVVNGATYDVSADGK

97-3250 KKASLITSETVYKNSAGLYTTTKVDNKAATLSDLDLNAAKKTGSTLVVNGATYDVSADGK

DEC10D KKASLITSETVYKNSAGLYTTTKVDNKAATLSDLDLNAAKKTGSTLVVNGATYDVSADGK

DEC10C KKASLITSETVYKNSAGLYTTTKVDNKAATLSDLDLNAAKKTGSTLVVNGATYDVSADGK

DEC10B KKASLITSETVYKNSAGLYTTTKVDNKAATLSDLDLNAAKKTGSTLVVNGATYDVSADGK

DEC9E KKASLITSETVYKNSAGLYTTTKVDNKAATLSDLDLNAAKKTGSTLVVNGATYDVSADGK

DEC9D KKASLITSETVYKNSAGLYTTTKVDNKAATLSDLDLNAAKKTGSTLVVNGATYDVSADGK

DEC9A KKASLITSETVYKNSAGLYTTTKVDNKAATLSDLDLNAAKKTGSTLVVNGATYDVSADGK

36348 KKASLITSETVYKNSAGLYTTTKVDNKAATLSDLDLNAAKKTGSTLVVNGATYDVSADGK

************************************************************

**BA2103 TITETASGNNKVMYLSKSEGGSPILVNEDAAKSLQSTTNPLETIDKALAKVDNLRSDLGA**

CFSAN001629 TITETASGNNKVMYLSKSEGGSPILVNEDAAKSLQSTTNPLETIDKALAKVDNLRSDLGA

CVM10030 TITETASGNNKVMYLSKSEGGSPILVNEDAAKSLQSTTNPLETIDKALAKVDNLRSDLGA

CVM10021 TITETASGNNKVMYLSKSEGGSPILVNEDAAKSLQSTTNPLETIDKALAKVDNLRSDLGA

CVM10224 TITETASGNNKVMYLSKSEGGSPILVNEDAAKSLQSTTNPLETIDKALAKVDNLRSDLGA

CVM9942 TITETASGNNKVMYLSKSEGGSPILVNEDAAKSLQSTTNPLETIDKALAKVDNLRSDLGA

CVM10026 TITETASGNNKVMYLSKSEGGSPILVNEDAAKSLQSTTNPLETIDKALAKVDNLRSDLGA

36079 TITETASGNNKVMYLSKSEGGSPILVNEDAAKSLQSTTNPLETIDKALAKVDNLRSDLGA

36084 TITETASGNNKVMYLSKSEGGSPILVNEDAAKSLQSTTNPLETIDKALAKVDNLRSDLGA

36943 TITETASGNNKVMYLSKSEGGSPILVNEDAAKSLQSTTNPLETIDKALAKVDNLRSDLGA

36293 TITETASGNNKVMYLSKSEGGSPILVNEDAAKSLQSTTNPLETIDKALAKVDNLRSDLGA

36708 TITETASGNNKVMYLSKSEGGSPILVNEDAAKSLQSTTNPLETIDKALAKVDNLRSDLGA

34870 TITETASGNNKVMYLSKSEGGSPILVNEDAAKSLQSTTNPLETIDKALAKVDNLRSDLGA

34827 TITETASGNNKVMYLSKSEGGSPILVNEDAAKSLQSTTNPLETIDKALAKVDNLRSDLGA

2011C-3274 TITETASGNNKVMYLSKSEGGSPILVNEDAAKSLQSTTNPLETIDKALAKVDNLRSDLGA

2010C-4244 TITETASGNNKVMYLSKSEGGSPILVNEDAAKSLQSTTNPLETIDKALAKVDNLRSDLGA

2010C-3902 TITETASGNNKVMYLSKSEGGSPILVNEDAAKSLQSTTNPLETIDKALAKVDNLRSDLGA

2010C-3871 TITETASGNNKVMYLSKSEGGSPILVNEDAAKSLQSTTNPLETIDKALAKVDNLRSDLGA

2010C-3472 TITETASGNNKVMYLSKSEGGSPILVNEDAAKSLQSTTNPLETIDKALAKVDNLRSDLGA

2010C-3051 TITETASGNNKVMYLSKSEGGSPILVNEDAAKSLQSTTNPLETIDKALAKVDNLRSDLGA

2009C-4826 TITETASGNNKVMYLSKSEGGSPILVNEDAAKSLQSTTNPLETIDKALAKVDNLRSDLGA

2009C-4760 TITETASGNNKVMYLSKSEGGSPILVNEDAAKSLQSTTNPLETIDKALAKVDNLRSDLGA

2009C-3996 TITETASGNNKVMYLSKSEGGSPILVNEDAAKSLQSTTNPLETIDKALAKVDNLRSDLGA

2009C-3612 TITETASGNNKVMYLSKSEGGSPILVNEDAAKSLQSTTNPLETIDKALAKVDNLRSDLGA

2009C-3689 TITETASGNNKVMYLSKSEGGSPILVNEDAAKSLQSTTNPLETIDKALAKVDNLRSDLGA

2011C-3655 TITETASGNNKVMYLSKSEGGSPILVNEDAAKSLQSTTNPLETIDKALAKVDNLRSDLGA

2011C-3282 TITETASGNNKVMYLSKSEGGSPILVNEDAAKSLQSTTNPLETIDKALAKVDNLRSDLGA

2011C-3506 TITETASGNNKVMYLSKSEGGSPILVNEDAAKSLQSTTNPLETIDKALAKVDNLRSDLGA

2011C-3387 TITETASGNNKVMYLSKSEGGSPILVNEDAAKSLQSTTNPLETIDKALAKVDNLRSDLGA

2011C-3270 TITETASGNNKVMYLSKSEGGSPILVNEDAAKSLQSTTNPLETIDKALAKVDNLRSDLGA

2010EL-1699 TITETASGNNKVMYLSKSEGGSPILVNEDAAKSLQSTTNPLETIDKALAKVDNLRSDLGA

2010C-5028 TITETASGNNKVMYLSKSEGGSPILVNEDAAKSLQSTTNPLETIDKALAKVDNLRSDLGA

2010C-4834 TITETASGNNKVMYLSKSEGGSPILVNEDAAKSLQSTTNPLETIDKALAKVDNLRSDLGA

2010C-4819 TITETASGNNKVMYLSKSEGGSPILVNEDAAKSLQSTTNPLETIDKALAKVDNLRSDLGA

2010C-4430 TITETASGNNKVMYLSKSEGGSPILVNEDAAKSLQSTTNPLETIDKALAKVDNLRSDLGA

03-3500 TITETASGNNKVMYLSKSEGGSPILVNEDAAKSLQSTTNPLETIDKALAKVDNLRSDLGA

06-3464 TITETASGNNKVMYLSKSEGGSPILVNEDAAKSLQSTTNPLETIDKALAKVDNLRSDLGA

05-3646 TITETASGNNKVMYLSKSEGGSPILVNEDAAKSLQSTTNPLETIDKALAKVDNLRSDLGA

11368 TITETASGNNKVMYLSKSEGGSPILVNEDAAKSLQSTTNPLETIDKALAKVDNLRSDLGA

97-3250 TITETASGNNKVMYLSKSEGGSPILVNEDAAKSLQSTTNPLETIDKALAKVDNLRSDLGA

DEC10D TITETASGNNKVMYLSKSEGGSPILVNEDAAKSLQSTTNPLETIDKALAKVDNLRSDLGA

DEC10C TITETASGNNKVMYLSKSEGGSPILVNEDAAKSLQSTTNPLETIDKALAKVDNLRSDLGA

DEC10B TITETASGNNKVMYLSKSEGGSPILVNEDAAKSLQSTTNPLETIDKALAKVDNLRSDLGA

DEC9E TITETASGNNKVMYLSKSEGGSPILVNEDAAKSLQSTTNPLETIDKALAKVDNLRSDLGA

DEC9D TITETASGNNKVMYLSKSEGGSPILVNEDAAKSLQSTTNPLETIDKALAKVDNLRSDLGA

DEC9A TITETASGNNKVMYLSKSEGGSPILVNEDAAKSLQSTTNPLETIDKALAKVDNLRSDLGA

36348 TITETASGNNKVMYLSKSEGGSPILVNEDAAKSLQSTTNPLETIDKALAKVDNLRSDLGA

************************************************************

**BA2103 VQNRFDSAITNLGNTVNNLSSARSRIEDADYATEVSNMSRAQILQQAGTSVLAQANQTTQ**

CFSAN001629 VQNRFDSAITNLGNTVNNLSSARSRIEDADYATEVSNMSRAQILQQAGTSVLAQANQTTQ

CVM10030 VQNRFDSAITNLGNTVNNLSSARSRIEDADYATEVSNMSRAQILQQAGTSVLAQANQTTQ

CVM10021 VQNRFDSAITNLGNTVNNLSSARSRIEDADYATEVSNMSRAQILQQAGTSVLAQANQTTQ

CVM10224 VQNRFDSAITNLGNTVNNLSSARSRIEDADYATEVSNMSRAQILQQAGTSVLAQANQTTQ

CVM9942 VQNRFDSAITNLGNTVNNLSSARSRIEDADYATEVSNMSRAQILQQAGTSVLAQANQTTQ

CVM10026 VQNRFDSAITNLGNTVNNLSSARSRIEDADYATEVSNMSRAQILQQAGTSVLAQANQTTQ

36079 VQNRFDSAITNLGNTVNNLSSARSRIEDADYATEVSNMSRAQILQQAGTSVLAQANQTTQ

36084 VQNRFDSAITNLGNTVNNLSSARSRIEDADYATEVSNMSRAQILQQAGTSVLAQANQTTQ

36943 VQNRFDSAITNLGNTVNNLSSARSRIEDADYATEVSNMSRAQILQQAGTSVLAQANQTTQ

36293 VQNRFDSAITNLGNTVNNLSSARSRIEDADYATEVSNMSRAQILQQAGTSVLAQANQTTQ

36708 VQNRFDSAITNLGNTVNNLSSARSRIEDADYATEVSNMSRAQILQQAGTSVLAQANQTTQ

34870 VQNRFDSAITNLGNTVNNLSSARSRIEDADYATEVSNMSRAQILQQAGTSVLAQANQTTQ

34827 VQNRFDSAITNLGNTVNNLSSARSRIEDADYATEVSNMSRAQILQQAGTSVLAQANQTTQ

2011C-3274 VQNRFDSAITNLGNTVNNLSSARSRIEDADYATEVSNMSRAQILQQAGTSVLAQANQTTQ

2010C-4244 VQNRFDSAITNLGNTVNNLSSARSRIEDADYATEVSNMSRAQILQQAGTSVLAQANQTTQ

2010C-3902 VQNRFDSAITNLGNTVNNLSSARSRIEDADYATEVSNMSRAQILQQAGTSVLAQANQTTQ

2010C-3871 VQNRFDSAITNLGNTVNNLSSARSRIEDADYATEVSNMSRAQILQQAGTSVLAQANQTTQ

2010C-3472 VQNRFDSAITNLGNTVNNLSSARSRIEDADYATEVSNMSRAQILQQAGTSVLAQANQTTQ

2010C-3051 VQNRFDSAITNLGNTVNNLSSARSRIEDADYATEVSNMSRAQILQQAGTSVLAQANQTTQ

2009C-4826 VQNRFDSAITNLGNTVNNLSSARSRIEDADYATEVSNMSRAQILQQAGTSVLAQANQTTQ

2009C-4760 VQNRFDSAITNLGNTVNNLSSARSRIEDADYATEVSNMSRAQILQQAGTSVLAQANQTTQ

2009C-3996 VQNRFDSAITNLGNTVNNLSSARSRIEDADYATEVSNMSRAQILQQAGTSVLAQANQTTQ

2009C-3612 VQNRFDSAITNLGNTVNNLSSARSRIEDADYATEVSNMSRAQILQQAGTSVLAQANQTTQ

2009C-3689 VQNRFDSAITNLGNTVNNLSSARSRIEDADYATEVSNMSRAQILQQAGTSVLAQANQTTQ

2011C-3655 VQNRFDSAITNLGNTVNNLSSARSRIEDADYATEVSNMSRAQILQQAGTSVLAQANQTTQ

2011C-3282 VQNRFDSAITNLGNTVNNLSSARSRIEDADYATEVSNMSRAQILQQAGTSVLAQANQTTQ

2011C-3506 VQNRFDSAITNLGNTVNNLSSARSRIEDADYATEVSNMSRAQILQQAGTSVLAQANQTTQ

2011C-3387 VQNRFDSAITNLGNTVNNLSSARSRIEDADYATEVSNMSRAQILQQAGTSVLAQANQTTQ

2011C-3270 VQNRFDSAITNLGNTVNNLSSARSRIEDADYATEVSNMSRAQILQQAGTSVLAQANQTTQ

2010EL-1699 VQNRFDSAITNLGNTVNNLSSARSRIEDADYATEVSNMSRAQILQQAGTSVLAQANQTTQ

2010C-5028 VQNRFDSAITNLGNTVNNLSSARSRIEDADYATEVSNMSRAQILQQAGTSVLAQANQTTQ

2010C-4834 VQNRFDSAITNLGNTVNNLSSARSRIEDADYATEVSNMSRAQILQQAGTSVLAQANQTTQ

2010C-4819 VQNRFDSAITNLGNTVNNLSSARSRIEDADYATEVSNMSRAQILQQAGTSVLAQANQTTQ

2010C-4430 VQNRFDSAITNLGNTVNNLSSARSRIEDADYATEVSNMSRAQILQQAGTSVLAQANQTTQ

03-3500 VQNRFDSAITNLGNTVNNLSSARSRIEDADYATEVSNMSRAQILQQAGTSVLAQANQTTQ

06-3464 VQNRFDSAITNLGNTVNNLSSARSRIEDADYATEVSNMSRAQILQQAGTSVLAQANQTTQ

05-3646 VQNRFDSAITNLGNTVNNLSSARSRIEDADYATEVSNMSRAQILQQAGTSVLAQANQTTQ

11368 VQNRFDSAITNLGNTVNNLSSARSRIEDADYATEVSNMSRAQILQQAGTSVLAQANQTTQ

97-3250 VQNRFDSAITNLGNTVNNLSSARSRIEDADYATEVSNMSRAQILQQAGTSVLAQANQTTQ

DEC10D VQNRFDSAITNLGNTVNNLSSARSRIEDADYATEVSNMSRAQILQQAGTSVLAQANQTTQ

DEC10C VQNRFDSAITNLGNTVNNLSSARSRIEDADYATEVSNMSRAQILQQAGTSVLAQANQTTQ

DEC10B VQNRFDSAITNLGNTVNNLSSARSRIEDADYATEVSNMSRAQILQQAGTSVLAQANQTTQ

DEC9E VQNRFDSAITNLGNTVNNLSSARSRIEDADYATEVSNMSRAQILQQAGTSVLAQANQTTQ

DEC9D VQNRFDSAITNLGNTVNNLSSARSRIEDADYATEVSNMSRAQILQQAGTSVLAQANQTTQ

DEC9A VQNRFDSAITNLGNTVNNLSSARSRIEDADYATEVSNMSRAQILQQAGTSVLAQANQTTQ

36348 VQNRFDSAITNLGNTVNNLSSARSRIEDADYATEVSNMSRAQILQQAGTSVLAQANQTTQ

************************************************************

**BA2103 NVLSLLR**

CFSAN001629 NVLSLLR

CVM10030 NVLSLLR

CVM10021 NVLSLLR

CVM10224 NVLSLLR

CVM9942 NVLSLLR

CVM10026 NVLSLLR

36079 NVLSLLR

36084 NVLSLLR

36943 NVLSLLR

36293 NVLSLLR

36708 NVLSLLR

34870 NVLSLLR

34827 NVLSLLR

2011C-3274 NVLSLLR

2010C-4244 NVLSLLR

2010C-3902 NVLSLLR

2010C-3871 NVLSLLR

2010C-3472 NVLSLLR

2010C-3051 NVLSLLR

2009C-4826 NVLSLLR

2009C-4760 NVLSLLR

2009C-3996 NVLSLLR

2009C-3612 NVLSLLR

2009C-3689 NVLSLLR

2011C-3655 NVLSLLR

2011C-3282 NVLSLLR

2011C-3506 NVLSLLR

2011C-3387 NVLSLLR

2011C-3270 NVLSLLR

2010EL-1699 NVLSLLR

2010C-5028 NVLSLLR

2010C-4834 NVLSLLR

2010C-4819 NVLSLLR

2010C-4430 NVLSLLR

03-3500 NVLSLLR

06-3464 NVLSLLR

05-3646 NVLSLLR

11368 NVLSLLR

97-3250 NVLSLLR

DEC10D NVLSLLR

DEC10C NVLSLLR

DEC10B NVLSLLR

DEC9E NVLSLLR

DEC9D NVLSLLR

DEC9A NVLSLLR

36348 NVLSLLR

*******
