# Supplementary material for: Flagellin and GroEL mediates in vitro binding of an atypical enteropathogenic Escherichia coli to cellular fibronectin
Source: BMC Microbiol. 2015 Dec 18;15:278. doi: 10.1186/s12866-015-0612-4 (PMC4683701; doi:10.1186/s12866-015-0612-4)
Supplement: Additional file 2: Figure S2. — Sequence alignment of the hypervariable region of aEPEC BA2103 flagellin and the fibronectin-binding region of Staphylococcus aureus FnBPA. A. Predicted amino acid sequence of aEPEC BA2103 flagellin (FliCBA2103), highlighting the hypervariable region (yellow). B. Amino acid sequence of S. aureus FnBPA, highlighting the Fn-binding region (grey). C. BLASTp results showing a 14-aa sequence of FliCBA2103 (residues 198 to 211 - query) with 64 % of similarity to two motifs of FnBPA (residues 764 to 777 and 802 to 815 - subject). D. Multiple sequence alignment of these three regions highlighting the conserved (dark grey) and similar (grey) amino acid residues. (DOCX 167 kb) [file 12866_2015_612_MOESM2_ESM.docx]

**A**

**>FliC_BA2103_**

1 MAQVINTNSLSLLTQNNLNKSQSSLSSAIERLSSGLRINSAKDDAAGQAIANRFTANIKG 60

61 LTQASRNANDGISVAQTTEGALNEINNNLQRVRELTVQATNGTNSDSDLSSIQAEITQRL 120

121 EEIDRVSEQTQFNGVKVLAENNEMKIQVGANDGETITINLAKIDAKTLGLDGFNIDGAQK 180

181 ATGSDLISKFKATGTDNYDVGGDAYTVNVDSGAVKDTTGNDIFVSAADGSLTTKSDTNIA 240

241 GTGIDATALAAAAKNKAQNDKFTFNGVEFTTTTAADGNGNGVYSAEIDGKSVTFTVTDAD 300

301 KKASLITSETVYKNSAGLYTTTKVDNKAATLSDLDLNAAKKTGSTLVVNGATYDVSADGK 360

361 TITETASGNNKVMYLSKSEGGSPILVNEDAAKSLQSTTNPLETIDKALAKVDNLRSDLGA 420

421 VQNRFDSAITNLGNTVNNLSSARSRIEDADYATEVSNMSRAQILQQAGTSVLAQANQTTQ 480

481 NVLSLLR 487

Hypervariable region (amino acids residues 177 to 394)

**B**

**>FnBPA (Fibronectin-binding Protein A – *Staphylococcus aureus*)**

1 MKNNLRYGIRKHKLGAASVFLGTMIVVGMGQDKEAAASEQKTTTVEENGNSATDNKTSET 60

61 QTTATNVNHIEETQSYNATVTEQPSNATQVTTEEAPKAVQAPQTAQPANIETVKEEVVKE 120

121 EAKPQVKETTQSQDNSGDQRQVDLTPKKATQNQVAETQVEVAQPRTASESKPRVTRSADV 180

181 AEAKEASNAKVETGTDVTSKVTVEIGSIEGHNNTNKVEPHAGQRAVLKYKLKFENGLHQG 240

241 DYFDFTLSNNVNTHGVSTARKVPEIKNGSVVMATGEVLEGGKIRYTFTNDIEDKVDVTAE 300

301 LEINLFIDPKTVQTNGNQTITSTLNEEQTSKELDVKYKDGIGNYYANLNGSIETFNKANN 360

361 RFSHVAFIKPNNGKTTSVTVTGTLMKGSNQNGNQPKVRIFEYLGNNEDIAKSVYANTTDT 420

421 SKFKEVTSNMSGNLNLQNNGSYSLNIENLDKTYVVHYDGEYLNGTDEVDFRTQMVGHPEQ 480

481 LYKYYYDRGYTLTWDNGLVLYSNKANGNEKNGPIIQNNKFEYKEDTIKETLTGQYDKNLV 540

541 TTVEEEYDSSTLDIDYHTAIDGGGGYVDGYIETIEETDSSAIDIDYHTAVDSEAGHVGGY 600

601 TESSEESNPIDFEESTHENSKHHADVVEYEEDTNPGGGQVTTESNLVEFDEESTKGIVTG 660

661 AVSDHTTVEDTKEYTTESNLIELVDELPEEHGQAQGPVEEITKNNHHISHSGLGTENGHG 720

721 NYDVIEEIEENSHVDIKSELGYEGGQNSGNQSFEEDTEEDKPKYEQGGNIVDIDFDSVPQ 780

781 IHGQNKGNQSFEEDTEKDKPKYEHGGNIIDIDFDSVPHIHGFNKHTEIIEEDTNKDKPSY 840

841 QFGGHNSVDFEEDTLPKVSGQNEGQQTIEEDTTPPIVPPTPPTPEVPSEPETPTPPTPEV 900

901 PSEPETPTPPTPEVPSEPETPTPPTPEVPAEPGKPVPPAKEEPKKPSKPVEQGKVVTPVI 960

961 EINEKVKAVAPTKKPQSKKSELPETGGEESTNKGMLFGGLFSILGLALLRRNKKNHKA 1018

FnBRs (Fibronectin-binding repeats) – residues 508 to 874

**C**

**BLASTp FliC_(177-394)_ vs. FnBPA_(508-874)_**

**
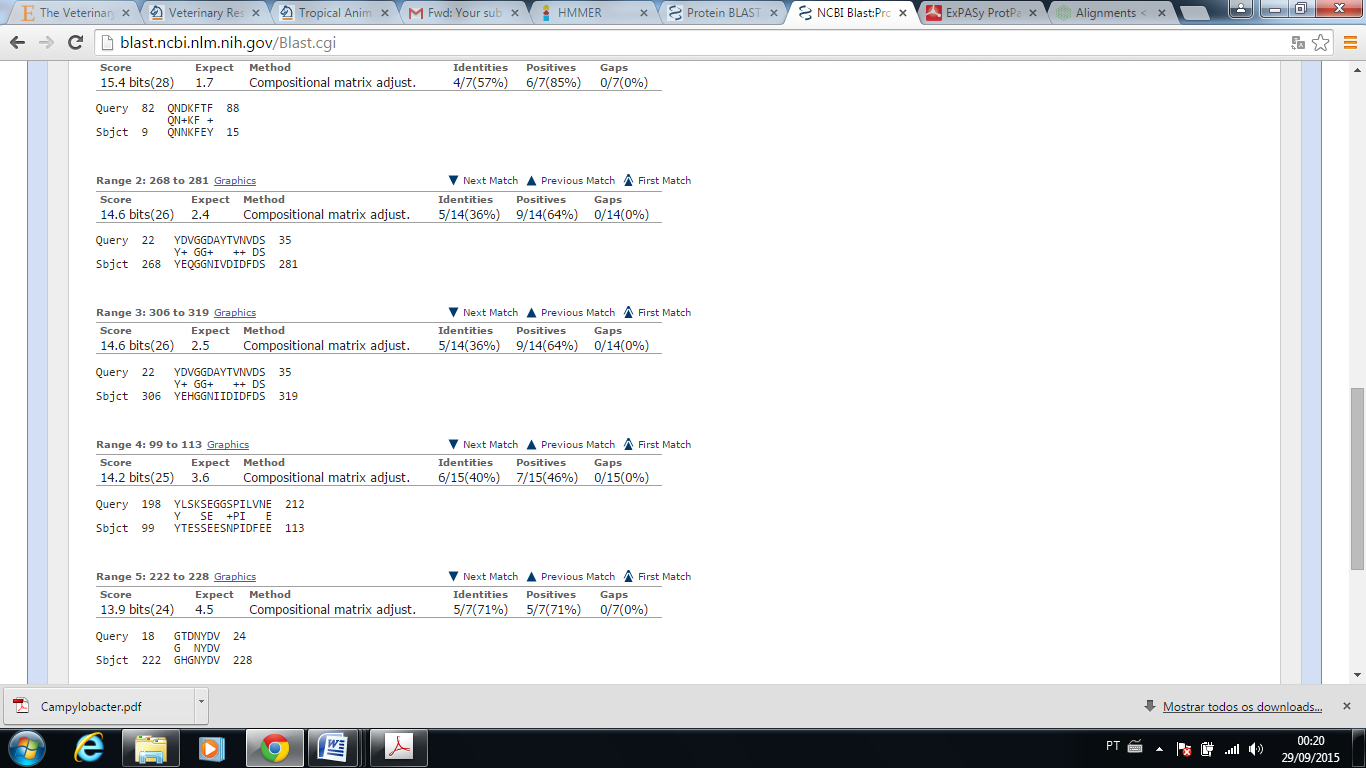

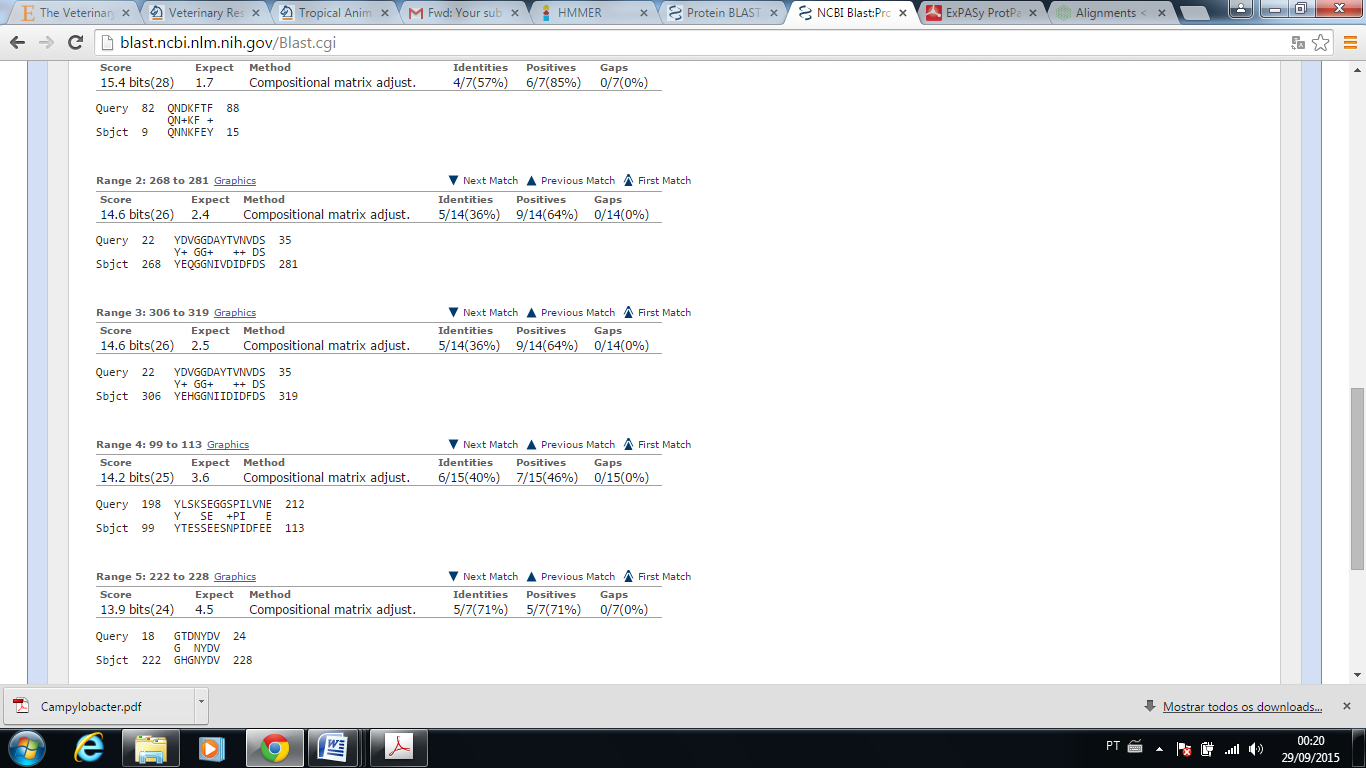
**

**D**

FnBPA(764-777) YEQGGNIVDIDFDS

FnBPA(802-815) YEHGGNIIDIDFDS

FliC(198-211) YDVGGDAYTVNVDS

*: **: :: **
