# Supplementary material for: Flagellin and GroEL mediates in vitro binding of an atypical enteropathogenic Escherichia coli to cellular fibronectin
Source: BMC Microbiol. 2015 Dec 18;15:278. doi: 10.1186/s12866-015-0612-4 (PMC4683701; doi:10.1186/s12866-015-0612-4)
Supplement: Additional file 3: Figure S3. — Amino acids sequences alignment of GroEL. The first sequence corresponding to E. coli J96 O4:K6 (gi|18028158), which was identified by MALDI-TOF of BA2103 isolated after fibronectin-associated immunoprecipitation. The sequence was compare to GroEL of Shigella sonnei 3233–85 (gi|391279593) and E. coli 11368 O26:H11 (gi|260853213:5396024–5397670) using BioEdit Sequence Alignment Editor vs 7.2.5.0 after alignment by ClustalW. The open box shows the amino acids differences among sequences at GroEL equatorial domain 1. The tagged sequence corresponding to conserved hydrophobic binding domain of GroEL. (DOCX 26 kb) [file 12866_2015_612_MOESM3_ESM.docx]

10 20 30 40 50 60 70

....|....|....|....|....|....|....|....|....|....|....|....|....|....|

**E. coli J96 O4:K6**  **-------------------------------------------MVFPLLVKS--------NWKNKFENMG**

**Shigella sonnei 3233-85**  **---------------MLRGVNVLADAVKVTLGPKGRNVVLDKSFGAPTITKDGVSVAREIELEDKFENMG**

**E. coli 11368 O26:H11**  **MAAKDVKFGNDARVKMLRGVNVLADAVKVTLGPKGRNVVLDKSFGAPTITKDGVSVAREIELEDKFENMG**

80 90 100 110 120 130 140

....|....|....|....|....|....|....|....|....|....|....|....|....|....|

**E. coli J96 O4:K6**  **AQMVKEVASKANDAAGDGTTTATVLAQAIITEGLKAVAAGMNPMDLKRGIDKAVTAAVEELKALSVPCSD**

**Shigella sonnei 3233-85**  **AQMVKEVASKANDAAGDGTTTATVLAQAIITEGLKAVAAGMNPMDLKRGIDKAVTAAVEELKALSVPCSD**

**E. coli 11368 O26:H11**  **AQMVKEVASKANDAAGDGTTTATVLAQAIITEGLKAVAAGMNPMDLKRGIDKAVTAAVEELKALSVPCSD**

150 160 170 180 190 200 210

....|....|....|....|....|....|....|....|....|....|....|....|....|....|

**E. coli J96 O4:K6**  **SKAIAQVGTISANSDETVGKLIAEAMDKVGKEGVITVEDGTGLQDELDVVEGMQFDRGYLSPYFINKPET**

**Shigella sonnei 3233-85**  **SKAIAQVGTISANSDETVGKLIAEAMDKVGKEGVITVEDGTGLQDELDVVEGMQFDRGYLSPYFINKPET**

**E. coli 11368 O26:H11**  **SKAIAQVGTISANSDETVGKLIAEAMDKVGKEGVITVEDGTGLQDELDVVEGMQFDRGYLSPYFINKPET**

220 230 240 250 260 270 280

....|....|....|....|....|....|....|....|....|....|....|....|....|....|

**E. coli J96 O4:K6**  **GAVELESPFILLADKKISNIREMLPVLEAVAKAGKPLLIIAEDVEGEALATAVVNTIRGIVKVAAVKAPG**

**Shigella sonnei 3233-85**  **GAVELESPFILLADKKISNIREMLPVLEAVAKAGKPLLIIAEDVEGEALATLVVNTMRGIVKVAAVKAPG**

**E. coli 11368 O26:H11**  **GAVELESPFILLADKKISNIREMLPVLEAVAKAGKPLLIIAEDVEGEALATLVVNTMRGIVKVAAVKAPG**

290 300 310 320 330 340 350

....|....|....|....|....|....|....|....|....|....|....|....|....|....|

**E. coli J96 O4:K6**  **FGDRRKAMLQDIATLTGGTVISEEIGMELEKATLEDLGQAKRVVINKDTTTIIDGVGEEAAIQGRVAQIR**

**Shigella sonnei 3233-85**  **FGDRRKAMLQDIATLTGGTVISEEIGMELEKATLEDLGQAKRVVINKDTTTIIDGVGEEAAIQGRVAQIR**

**E. coli 11368 O26:H11**  **FGDRRKAMLQDIATLTGGTVISEEIGMELEKATLEDLGQAKRVVINKDTTTIIDGVGEEAAIQGRVAQIR**

360 370 380 390 400 410 420

....|....|....|....|....|....|....|....|....|....|....|....|....|....|

**E. coli J96 O4:K6**  **QQIEEATSDYDREKLQERVAKLAGGVAVIKVGAATEVEMKEKKARVEDALHATRAAVEEGVVAGGGVALI**

**Shigella sonnei 3233-85**  **QQIEEATSDYDREKLQERVAKLAGGVAVIKVGAATEVEMKEKKARVEDALHATRAAVEEGVVAGGGVALI**

**E. coli 11368 O26:H11**  **QQIEEATSDYDREKLQERVAKLAGGVAVIKVGAATEVEMKEKKARVEDALHATRAAVEEGVVAGGGVALI**

430 440 450 460 470 480 490

....|....|....|....|....|....|....|....|....|....|....|....|....|....|

**E. coli J96 O4:K6**  **RVASKLADLRGQNEDQNVGIKVALRAMEAPLRQIVLNCGEEPSVVANTVKGGDGNYGYNAATEEYGNMID**

**Shigella sonnei 3233-85**  **RVASKLADLRGQNEDQNVGIKVALRAMEAPLRQIVLNCGEEPSVVANTVKGGDGNYGYNAATEEYGNMID**

**E. coli 11368 O26:H11**  **RVASKLADLRGQNEDQNVGIKVALRAMEAPLRQIVLNCGEEPSVVANTVKGGDGNYGYNAATEEYGNMID**

500 510 520 530 540

....|....|....|....|....|....|....|....|....|....|....|....

**E. coli J96 O4:K6**  **MGILDPTKVTRSALQYAASVAGLMITTECMVTDLPKNDAADLGAAGGMGGMGGMGGMM-**

**Shigella sonnei 3233-85**  **MGILDPTKVTRSALQYAASVAGLMITTECMVTDLPKNDAADLGAAGGMGGMGGMGGMM-**

**E. coli 11368 O26:H11**  **MGILDPTKVTRSALQYAASVAGLMITTECMVTDLPKNDAADLGAAGGMGGMGGMGGMMX**
