# Supplementary figures and images for: Flagellin and GroEL mediates in vitro binding of an atypical enteropathogenic Escherichia coli to cellular fibronectin
Source: BMC Microbiol. 2015 Dec 18;15:278. doi: 10.1186/s12866-015-0612-4 (PMC4683701; doi:10.1186/s12866-015-0612-4)

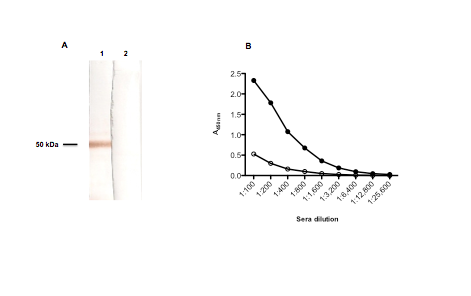

Supplement: Additional file 4: Figure S4. — Flagellin purification and characterization. A. Nitrocellulose membrane containing 10 μg of purified flagellin. Immunoblotting reaction was carried out using anti-H11 rabbit serum (1) and naïve rabbit serum (2), followed by goat IgG anti-rabbit peroxidase conjugate. Arrow indicates flagellin. B. Titration of anti-H11 and naïve rabbit sera by indirect ELISA. (TIFF 26 kb) [file 12866_2015_612_MOESM4_ESM.tiff]
